# Guiding functional near-infrared spectroscopy optode-layout design using individual (f)MRI data: effects on signal strength

**DOI:** 10.1117/1.NPh.8.2.025012

**Published:** 2021-06-17

**Authors:** Amaia Benitez-Andonegui, Michael Lührs, Laurien Nagels-Coune, Dimo Ivanov, Rainer Goebel, Bettina Sorger

**Affiliations:** a Maastricht University, Maastricht Brain Imaging Center, Department of Cognitive Neuroscience, Maastricht, The Netherlands; b Maastricht University, Laboratory for Cognitive Robotics and Complex Self-Organizing Systems, Department of Data Science and Knowledge Engineering, Maastricht, The Netherlands; c Brain Innovation B.V., Research Department, Maastricht, The Netherlands

**Keywords:** functional near-infrared spectroscopy, functional magnetic resonance imaging, optode layout design, mental imagery, brain–computer interfaces

## Abstract

**Significance:** Designing optode layouts is an essential step for functional near-infrared spectroscopy (fNIRS) experiments as the quality of the measured signal and the sensitivity to cortical regions-of-interest depend on how optodes are arranged on the scalp. This becomes particularly relevant for fNIRS-based brain–computer interfaces (BCIs), where developing robust systems with few optodes is crucial for clinical applications.

**Aim:** Available resources often dictate the approach researchers use for optode-layout design. We investigated whether guiding optode layout design using different amounts of subject-specific magnetic resonance imaging (MRI) data affects the fNIRS signal quality and sensitivity to brain activation when healthy participants perform mental-imagery tasks typically used in fNIRS-BCI experiments.

**Approach:** We compared four approaches that incrementally incorporated subject-specific MRI information while participants performed mental-calculation, mental-rotation, and inner-speech tasks. The literature-based approach (LIT) used a literature review to guide the optode layout design. The probabilistic approach (PROB) employed individual anatomical data and probabilistic maps of functional MRI (fMRI)-activation from an independent dataset. The individual fMRI (iFMRI) approach used individual anatomical and fMRI data, and the fourth approach used individual anatomical, functional, and vascular information of the same subject (fVASC).

**Results:** The four approaches resulted in different optode layouts and the more informed approaches outperformed the minimally informed approach (LIT) in terms of signal quality and sensitivity. Further, PROB, iFMRI, and fVASC approaches resulted in a similar outcome.

**Conclusions:** We conclude that additional individual MRI data lead to a better outcome, but that not all the modalities tested here are required to achieve a robust setup. Finally, we give preliminary advice to efficiently using resources for developing robust optode layouts for BCI and neurofeedback applications.

## 1 Introduction

Functional near-infrared spectroscopy (fNIRS) is a noninvasive, portable optical imaging method used to measure brain activity via hemodynamic responses involving increased oxygen consumption and cerebral blood flow.^1^^–^^3^ These physiological changes lead to local changes in the concentrations of oxy- (Δ[HbO]) and deoxyhemoglobin (Δ[HbR]), which can be detected because near-infrared light is absorbed by hemoglobin located in blood vessels.^3^^,^^4^

When setting up an fNIRS experiment, optodes are placed on the scalp, which can be classified into sources (emitters) and detectors (receivers) depending on their function. Light emitted from a source is propagated through extracerebral and cerebral tissues up to a few centimeters, where some photons are scattered and absorbed before light reaches the detectors.^5^ The spatial resolution of fNIRS is therefore in the range of 5 to 10 mm^4^ depending on the way source–detector pairs (or “channels”) are arranged on the scalp.^6^ The distance between a source and detector pair along with the anatomical tissues between them determines the depth of light penetration and the sensitivity to underlying cortex.^1^ Therefore, the quality of the fNIRS signal can differ dramatically between optode layouts.

This effect of optode layout is particularly relevant for applications requiring sparse optode layouts, such as brain–computer interfaces (BCIs). BCIs provide an alternative means of motor-independent communication for clinical populations suffering from severe motor disabilities^7^ by enabling users to send commands via brain activity in the absence of motor output.^7^^,^^8^ FNIRS is a promising choice for implementing BCIs due to its portability, safety, and relatively low cost.^9^^,^^10^ However, it remains a challenging undertaking to develop efficient, accurate, and robust systems using the limited number of optodes required for fNIRS-BCI systems to remain portable and comfortable for clinical applications. Indeed, a number of fNIRS-based BCI studies using sparse (<14 channels) and localized optode layouts^11^^–^^16^ reported variability in the accuracy reached by participants (16.67% to 100% accuracy for two-class problems, 46.5% to 66.8% for a three-class problem, and 37.5% to 100% for a six-class problem). This variability may originate from individual anatomical^17^^,^^18^ or functional differences^15^ that affect fNIRS signal quality/sensitivity and therefore might be improved by designing optode layouts for individual users that account for such differences.

Researchers often define a region-of-interest (ROI) in line with their research question and design an optode layout in a grid-like fashion to target a specific brain area.^1^ The simplest and most common optode-layout design is to assign source and detector locations on the head to cover a given cortical ROI according to the standardized 10-20 electroencephalography (EEG) system or its extended versions.^19^ These locations can be related to the underlying assumed cortical structure^20^^,^^21^ or to the standard Montreal Neurological Institute (MNI) stereotactic coordinates.^22^^–^^25^ This procedure has proven effective for many applications but may be suboptimal for use in BCIs. In this study, we were interested in whether incorporating additional neuroimaging data such as anatomical or functional magnetic resonance imaging (MRI or fMRI) can improve optode-layout design for use in BCIs.

The selection of the ROIs in the procedure described above is commonly based on anatomically defined coordinates only. However, ROIs derived from functional neuroimaging techniques such as fMRI could increase the spatial specificity of ROI definition by accounting for individual local differences in elicited brain activity for a given task. Once an ROI is defined, the fNIRS community has developed several approaches to optimize optode-layout designs using light-sensitivity profiles.^1^ Light-sensitivity profiles are probabilistic models of photon absorption based on the tissues found between source and detector optodes.^26^ Software packages, toolboxes, and pipelines compute these profiles using Monte Carlo simulations to optimize optode layouts,^1^^,^^5^^,^^26^^–^^29^ thus promising an increase on signal quality and sensitivity for BCI applications. However, light sensitivity profile models require anatomical head data, either from an MRI-derived atlas or from subject-specific MRI data. MRI atlases are an appealing option for computing profiles, as they do not require additional MRI measurements, which may be expensive, time-consuming, or generally unavailable. That said, subject-specific MRI data better capture specific anatomical and vascular features and therefore could improve the robustness of fNIRS setups across individuals. Considering subject-specific vascular information may be particularly relevant, as vascular structures are highly scattering and absorbing media^30^ and can influence the estimates of light sensitivity profiles.^31^

Naturally, available resources for collecting additional data dictate the approach researchers use to design optode layouts. We therefore asked the following question: What is the potential gain of incorporating (anatomical, functional, and vascular) MRI data when optimizing optode-layout designs for fNIRS-based BCIs? With this question in mind, we selected four approaches that incrementally incorporated the amount of individual information from the same participant to design subject-specific optode layouts. The first layout was the literature-based approach (hereinafter referred to as LIT), where optodes were selected based on a literature review. LIT represents the scenario where no additional individual MRI information is available. The second setup was the probabilistic approach (referred to as PROB), which employed individual anatomical data together with a probabilistic functional map derived from an independent dataset to inform optode placement. PROB illustrates a situation where individual fMRI data are not available, but subject-specific anatomical information and functional data from other individuals are accessible. The third setup was the individual fMRI approach, which used anatomical data and functional activation maps of the same individual (referred to as iFMRI). Finally, the fourth setup was the vascular approach, which used individual anatomical, functional, and vascular information of the same subject (referred to as fVASC).

We assessed whether different approaches resulted in distinct optode layouts and assessed whether the quality of the fNIRS signal and the detected task-related activation (fNIRS sensitivity) differed across optode layouts. Participants were asked to perform three mental-imagery tasks commonly used for hemodynamic BCIs, see Table S3 in the Supplementary Material: mental-calculation, mental-rotation, and inner-speech. We designed approach-specific optode layouts using Monte Carlo simulations and an algorithmic procedure that used two main constraints: (1) the interoptode distance did not exceed the 25 to 40 mm range to provide a reasonable signal-to-noise ratio (SNR)^32^ and (2) the optode layout for each approach consisted of two channels that shared a common source. Restricting the layout to two channels was motivated by its suitability in clinical settings due to its easy setup and participant comfort, and by the abovementioned BCI studies that showed encouraging results using small setups. In addition, this constraint allowed us to compare the four approaches within the same functional fNIRS run. We hypothesized that each approach would lead to different optode-layout designs and that the SNR of resulting fNIRS signal would improve with more individualized approaches. Our results show that the four approaches indeed result in different optode layouts and that the more individualized approaches (PROB, iFMRI, and fVASC) outperform the minimally informed approach (LIT) in terms of fNIRS signal quality and sensitivity. Further, we find that PROB, iFMRI, and fVASC approaches produce similar signal quality and sensitivity. Finally, we give preliminary recommendations to help researchers efficiently use resources for developing robust and convenient optode layouts for fNIRS-BCIs.

## 2 Materials and Methods

**Fig. 1:**
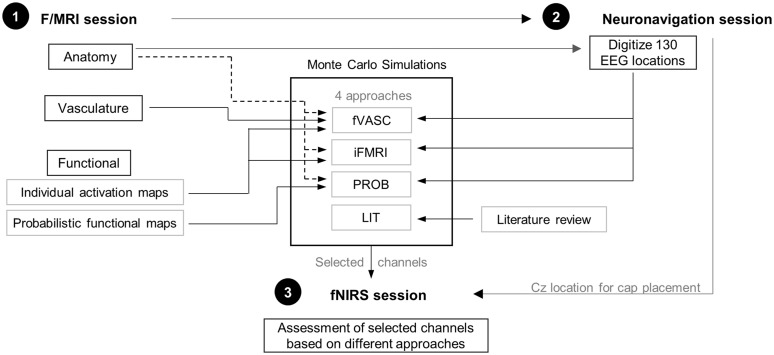
Overview of this study. The study consisted of three separate sessions: one f/MRI, one neuronavigation, and one fNIRS session. The first two sessions aimed at collecting necessary information to create the different optode layouts for each participant. Specifically, the LIT approach used a literature review to design the optode layout. The PROB approach used probabilistic functional MRI maps, individual anatomical data, and head-anatomy information for channel selection. The iFMRI approach used individual anatomical data and individual functional activation maps, together with head-anatomy information for channel selection. Finally, the fVASC approach used individual anatomical, functional, and vascular data, together with head-anatomy information for channel selection. Monte Carlo simulations were used to select the best channel pair for each approach, mental-imagery task and participant. The selected channels were used during the fNIRS session to obtain information on signal quality and to measure functional activity elicited by the mental-imagery tasks.

This experiment consisted of three separate sessions that took place in the following order: one f/MRI session, a neuronavigation session, and an fNIRS session. The first two sessions aimed at gathering necessary information for designing optode layouts, while the fNIRS session aimed at acquiring data to assess and compare the designed optode layouts (see Fig. 1).

Twenty-one participants (11 females) were recruited for the f/MRI session. From these participants, 17 (11 females) took part in the neuronavigation session and 16 (10 females) participated in the fNIRS session (see Table S1 in the Supplementary Material for a summary) as some participants became unavailable over the sessions. Participants did not have a history of neurological disease and had a normal or corrected-to-normal vision. The experiment conformed to the *Declaration of Helsinki* and was approved by the ethics committee of the *Faculty of Psychology and Neuroscience*, *Maastricht University*. Informed consent was obtained from each participant before starting the experiment. Participants received financial compensation after each session.

### 2.1 f/MRI Session

#### 2.1.1 Data acquisition

In this 1-h long session, anatomical, functional, and (brain and scalp) vascular data were acquired at a Siemens Magnetom Prisma Fit 3 Tesla (T) scanner at the Maastricht Brain Imaging Center, Maastricht, The Netherlands. Acquisition parameters are described in Sec. A.1 in the Supplementary Material.

#### 2.1.2 Experimental design

**Fig. 2:**
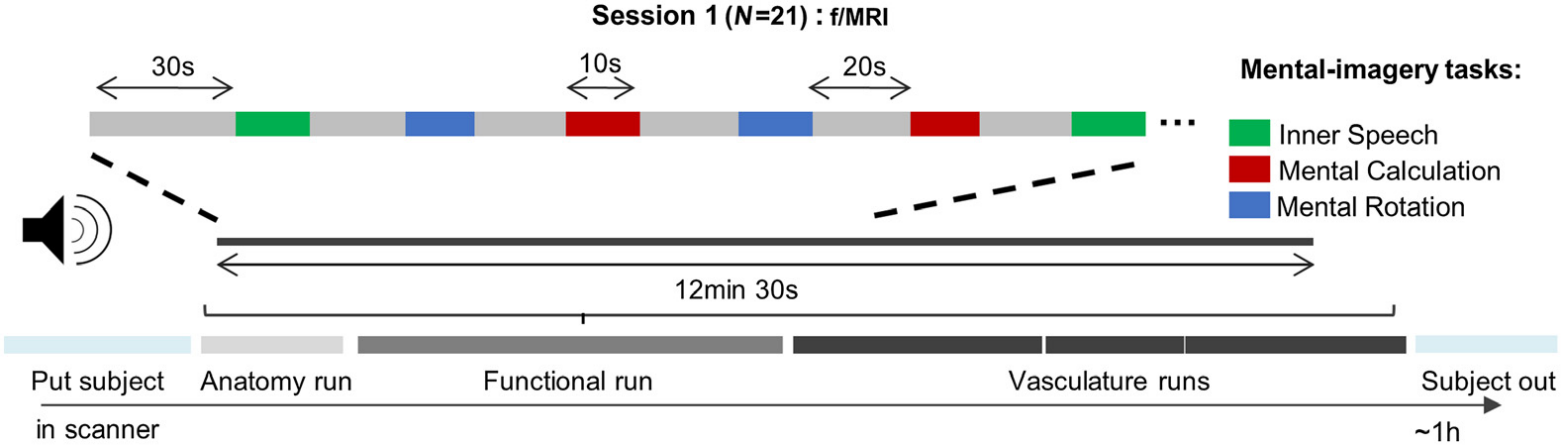
Schematic representation of session 1. Twenty-one participants underwent a 1-h long experiment in the MRI scanner, during which individual anatomical, functional, and vascular data were collected. During the functional run, participants had to perform inner-speech, mental-calculation, or mental-rotation for 10 s each with interleaved resting periods of 20 s. Task order was randomized.

Participants performed one ∼13-min long functional run, where they were acoustically cued to rest (Rest) or perform one of the three mental-imagery tasks, namely inner- (covert) speech (Speech), mental-calculation (Calculate), or mental-rotation (Rotate). The order of the task trials (eight trials per mental task) was randomized. They were instructed to covertly recite a text they knew by heart (e.g., a poem) when they heard “Speech.” Participants were asked to calculate multiplication tables of multiples of 7, 8, or 9 up to the decuple when they heard “Calculate.” When they heard “Rotate,” participants had to imagine a diver jumping from a tower into the water while (s)he spins around several times in the air. Participants were trained on the tasks for ∼10  min before entering the MRI scanner (see details in Sec. A1 in Supplementary Material). We instructed participants to perform the mental-imagery tasks, which lasted 10 s, until they heard the instruction “Rest.” During resting period, participants were asked not to do any specific mental activity and not to do or think about anything in particular for 20 s (see Fig. 2 for a visualization of the run). Participants kept their eyes closed throughout the functional run. After the session, participants’ strategies were noted down and saved for the fNIRS session (see Table S2 in the Supplementary Material for examples of such strategies). BrainStim v1.1.0.1 stimuli presentation software (Gijsen, S., Maastricht University, The Netherlands) was used for both the f/MRI and fNIRS sessions.

#### 2.1.3 Data analysis

This section will briefly describe the data analyses steps that were followed for each modality. For further details, we refer the reader to Sec. A.2 in the Supplementary Material. Unless stated otherwise, all f/MRI data analyses were performed in BrainVoyager QX v2.8 (Brain Innovation B.V., Maastricht, The Netherlands).

##### Structural and vascular data

Structural images were aligned to the plane containing the anterior and posterior commissures, corrected for spatial-intensity inhomogeneities and brain-masked. The white/gray matter (WM/GM) and gray matter/cerebrospinal (GM/CSF) boundaries were detected using automatic segmentation tools. These images were inspected, manually corrected when necessary, and used to create WM and GM reconstructions of the cortical surface. Vascular data were aligned to the anatomical data for each participant, segmented using automatic segmentation tools, and manually corrected when necessary. For more details, see Sec. A.2 in the Supplementary Material.

##### Functional data

Functional data were preprocessed and spatially coregistered to the structural image. Next, we calculated a voxelwise general linear model that contained a separate boxcar predictor for each of the mental-imagery task conditions convolved with a standard double-gamma hemodynamic response function and six additional predictors estimated from the motion-estimation procedure in BrainVoyager QX. Individual functional maps were created by contrasting each mental-imagery task versus the rest condition in the voxels that were part of the fNIRS-coverage mask (see Sec. A.2 and Fig. S1 in the Supplementary Material for details) and corrected using a cluster threshold that allowed for a 5% loss of active voxels. These functional maps were then sampled to surface activation maps (from −1 to +3  mm from the GM/WM segmentation boundary) for generating subject-specific probabilistic maps.

Probabilistic functional maps were created separately for each participant and mental-imagery task following a leave-one-subject-out procedure.^33^ In short, for each participant, surface activation maps from the remaining participants were first aligned to a common space (see Sec. A.2 in the Supplementary Material) and a probabilistic map was computed for each mental imagery task and hemisphere. The resulting maps were transformed back into individual volume space. The final maps (three per participant) were used as ROIs for Monte Carlo simulations (see Sec. 2.2.2). Examples of probabilistic maps are shown in Fig. S2 in the Supplementary Material.

##### Neuronavigation session

**Fig. 3:**
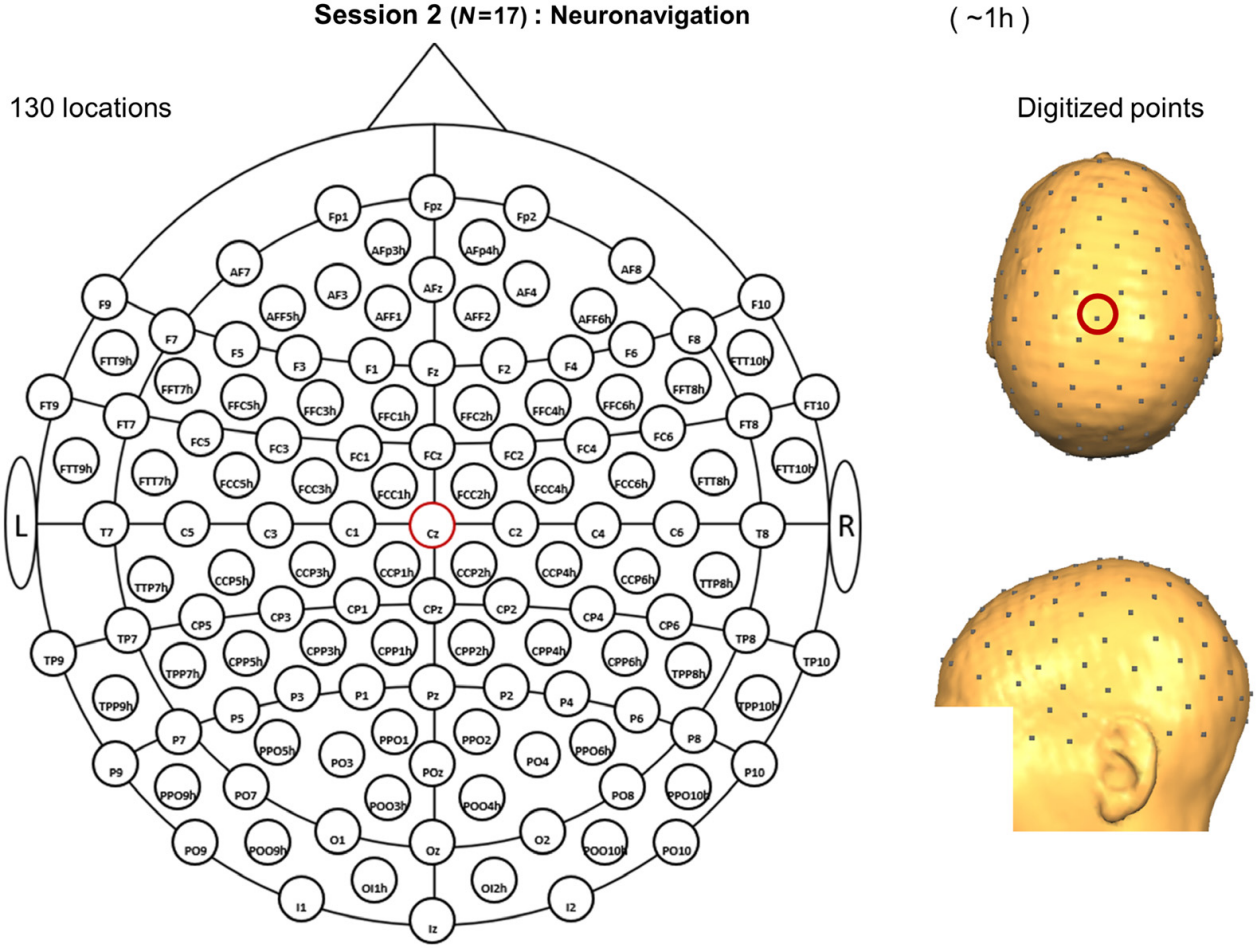
Schematic (left) and reconstructed (right) locations recorded during the neuronavigation session. This layout is an extension of the international 10-20 system, it contains 130 locations and the nomenclature is based on 19. The Cz location is indicated with a red circle. The schematic representation is based on the NIRx montage editor template, while the reconstructed locations belong to participant P04.

Seventeen of the originally included 21 participants underwent this session, as P07, P08, P13, and P18 dropped out of the study. A neuronavigation system (Zebris CMS20 ultrasound system, Zebris Medical GmbH, Isny, Germany) in combination with BrainVoyager QX 2.1 TMS Neuronavigator software (Brain Innovation, Maastricht, The Netherlands) was used to acquire the coordinates of 130 EEG positions for each participant (see Fig. 3). These 130 locations were determined based on the layout of EasyCap 128Ch ActiCap (EasyCap GmbH, Herrsching, Germany), whose size was selected based on individual head sizes. Specific details on cap and sensor placements can be found in Sec. A.3 in the Supplementary Material. The session lasted ∼1  h.

### 2.2 fNIRS Session

#### 2.2.1 Participants

P12 dropped out of the study. Thus, 16 of the 17 participants that participated in the f/MRI and neuronavigation sessions took part in this session, out of which 10 were female (mean age=29.81±5.22).

#### 2.2.2 Designing approach-specific optode layouts

**Fig. 4:**
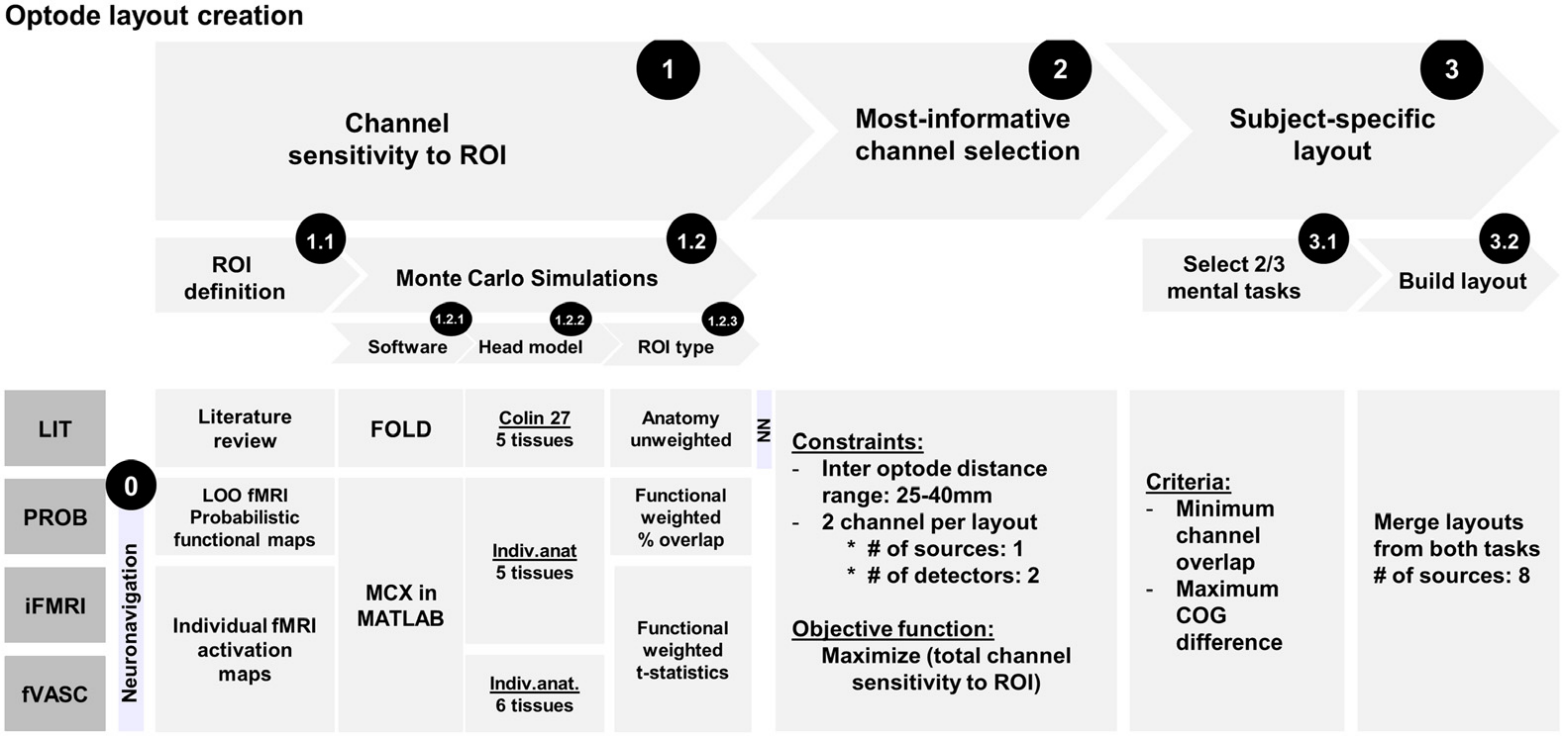
Summary of the key steps involved in optode-layout design for each of the four approaches evaluated in this study. The process was divided into three main stages: (1) channel sensitivity to ROI computation, (2) channel selection, and (3) building a subject-specific layout. For the first stage, each of the four approaches had a unique combination of ROI definition/type, software, and brain model used to compute the Monte Carlo simulations. During the second stage, the most-informative channels were selected for each of the four approaches and two mental-imagery tasks. The last stage combined all the layouts into one. LOO, leave-one-out; COG, center of gravity; NN, neuronavigation.

This process can be divided into three main stages: channel-sensitivity computation, channel selection, and building a participant-specific layout (see Fig. 4 for a summary).

##### Channel sensitivity to ROI computation

The first stage aimed at computing the channel-sensitivity profiles using Monte Carlo simulations. Each of the four approaches had a unique combination of ROI definition and type, software, and brain model used to compute the simulations.

The LIT approach represents a scenario where no individual MRI anatomical data are available, and the target ROI is selected based on a literature review. Given such scenario, fOLD toolbox^29^ provides an easy way to compute the sensitivity profiles to the selected ROIs. This is because fOLD uses an atlas head model as the input to the Monte Carlo simulation and offers different brain parcellation atlases for ROI definition in the target head-model space. In addition, it is freely available, easy to install, and has a user-friendly graphical interface. fOLD uses MCX package^34^ to compute the light-sensitivity profiles of optodes placed in predefined locations on the scalp, namely points corresponding to the extended 10-10 and 10-5 systems (130 points in total). It then provides a list of channels with the highest sensitivity to the ROI that can be exported for subsequent computations. PROB, iFMRI, and fVASC approaches represent scenarios where individual MRI anatomical data are accessible. Since fOLD does not offer the option of using individual head models to compute Monte Carlo simulations, these were computed using the MCX package directly through its MATLAB interface (v2015b, The MathWorks, Inc., Natick, Massachusetts). The remaining differences between the four approaches are summarized in Table 1, and more detailed information is provided in the Supplementary Material.

**Table 1:** Comparison between Monte Carlo simulation approaches.

	fOLD				DIRECT MCX			
Approach where software is used	LIT				PROB, iFMRI, fVASC			
Number of simulated photons	$10^{8}$							
Source modeling	Pencil source							
Detector modeling	Pencil source							
Source/detector locations	130 points according to extended 10-20 EEG systems (defined using Mesh2EEG^a^)				130 points according to extended 10-20 EEG system + subject-tailored (derived from neuronavigation session)			
Channel definition criterion	Neighboring optical positions on 10-10/10-5 systems (median interoptode distance of 36 mm)				Interoptode distance range of 20 to 45 mm			
Anatomical model	Atlas head model (MNI Colin 27)				Individual anatomy (individual space)			
Number of tissues	5				5 (PROB, iFMRI) or 6 (fVASC)			
Wavelength (nm)	Mean (690, 750, 780 830) [default in fOLD]							
Optical properties	Used?						Used?	
	LIT	Tissue	μs (${\mathrm{mm}}^{-1}$)	g	μa (${\mathrm{mm}}^{-1}$)	n	PROB, iFMRI	fVASC
	Yes	Scalp	0.72	0.01	0.017275	1	Yes	Yes
	Yes	Skull	0.92	0.01	0.011925	1	Yes	Yes
	Yes	CSF	0.01	0.01	0.002500	1	Yes	Yes
	Yes	Gray matter	1.10	0.01	0.019500	1	Yes	Yes
	Yes	White matter	1.35	0.01	0.016900	1	Yes	Yes
	No	Vasculature	73.31	0.405	0.9825	1	No	Yes
Resolution of anatomical model	2×2×2 mm				1×1×1 mm			
ROI type	Anatomical (literature review + Juelich brain parcellation)				Functionally derived			
Output type	Anatomical sensitivity (in %) to a given ROI				Functional sensitivity (in %) to a given ROI			
Platform for MCX simulations	Ubuntu 16.04.02 LTS (Xenial Xeurs) with Intel Xeon E52650 v3 2.3 GHz, GeForce Gtx 770, and CUDA 8.0				Ubuntu 16.04.4 LTS, Intel(R) Xeon(R) CPU E5-2697 v2 @ 2.70 GHz, 256 GB RAM, Tesla K20Xm, and CUDA 9.1.85			

a Multimodal Neuroimaging Laboratory; μs/g/μa/n: scattering/anisotropy/absorption/refraction parameters.

##### Optimization of the optode layout

During the second stage, the most-informative channels were selected for each of the four approaches and tasks by maximizing their total sensitivity to the target ROI. The maximization problem was subject to two constraints:

(1) The interoptode distance was limited to the 25- to 40-mm range.
(2) The optode layout for each approach consisted of two channels that shared a common detector (thus including three optodes per approach).

We followed an iterative approach to address the optimization problem. It begins with the construction of an empty solution, where no optode pair is selected. The algorithm then prunes the optode pairs that do not satisfy the interoptode distance range constraint. Next, it ranks all possible optode pairs according to their contribution to the total sensitivity and selects one pair as the seed in each iteration. The algorithm then transfers the selected optode pair to the solution matrix, and it removes from the list the channels that do not share the same detector. Next, it selects the first channel from this list (i.e., the one with the highest sensitivity). Since the target number of channels (=2) has been reached after this step, the accumulated total sensitivity of the selected two channels and the source–detector indices are stored in the solution matrix. These steps are repeated until all optode pairs are used as seeds. Finally, the two channels that lead to the highest total sensitivity for either constraint set constitute the selected channels for creating the setup.

##### Creating the setup

The first and second stages were repeated until approach- and task-specific optode layouts were created (12 per participant, since there were three tasks and four approaches). The last stage aimed at combining all optode layouts into a single one individually for each participant.

Two out of the three mental-imagery tasks that participants performed during the f/MRI session were selected for the fNIRS session. This measure was necessary as pilot measurements performed with optode layouts designed to account for all three tasks elicited high discomfort in participants. This decision ensured that the optode setup would maximally consist of 24 optodes (three optodes per layout × four approaches × two mental-imagery tasks), which should constitute a reasonably comfortable setup for participants and thus should prevent them from withdrawing from fNIRS recordings due to setup-related discomfort.^35^^–^^37^ This selection was carried out at the individual subject level. Finally, the eight layouts (four per task) were combined manually into a single one. See Table S7 in the Supplementary Material for a summary of the mental-imagery task selection procedure and Table S8 in the Supplementary Material for the resulting selected task pair per participant.

#### 2.2.3 Experimental design

**Fig. 5:**
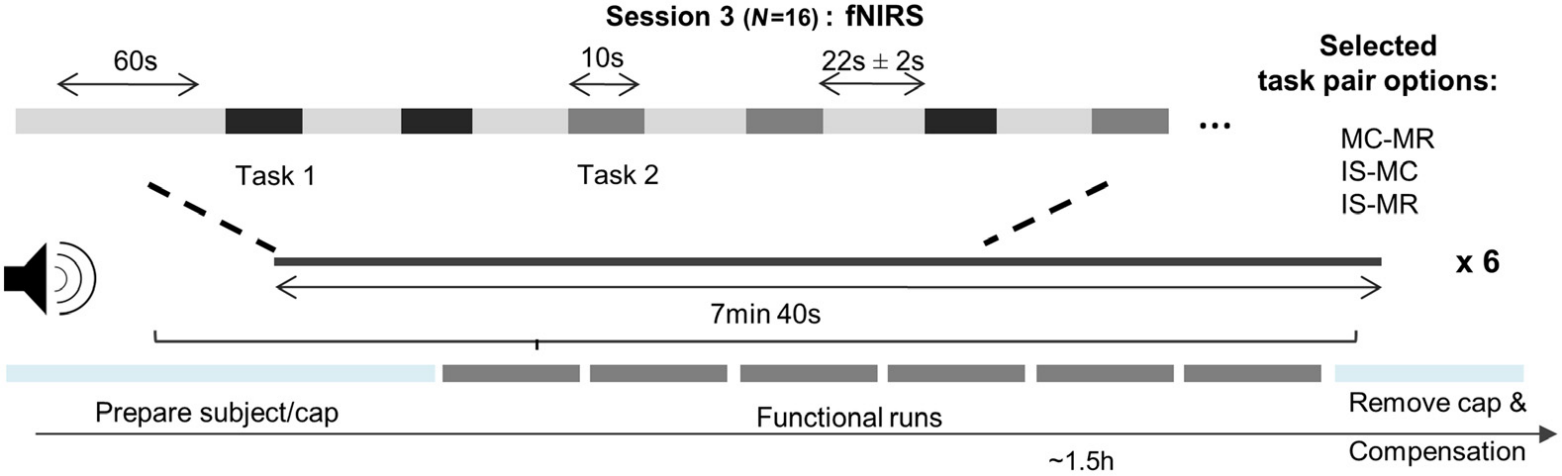
Schematic representation of a functional run during the fNIRS session. During each mental-task period, participants were acoustically cued to perform one of the two mental-imagery tasks for 10 s while keeping their eyes closed. When participants heard “rest,” they were asked to stop the task and await the next instruction. IS, inner-speech; MC, mental-calculation; MR, mental-rotation.

The fNIRS experiment consisted of one session that lasted ∼1.5  h. During this time, participants performed six, around 8-min long functional runs. In each of the runs, participants were acoustically cued to perform one of the two mental-imagery tasks selected for them or to rest. Six, 10-s long trials were presented for each mental-imagery task, interleaved with a jittered rest condition with mean duration of 22 s (jittering was of ±2  s), see Fig. 5. Thus, participants performed 60 trials for each mental-imagery task across the six runs. Trials were pseudorandomized across runs. Participants were instructed to use the same strategy they used in the scanner (first session). For that, they were given a document prior to the fNIRS experiment where their strategies had been noted down. Participants were asked to avoid any potential jaw movements during the functional runs and to keep their eyes closed throughout the run.

#### 2.2.4 fNIRS signal acquisition

fNIRS data were recorded using a continuous-wave system (NIRScout-816, NIRx, Medizintechnik GmbH, Berlin, Germany). The optode setup varied across participants, but they had some features in common: all setups contained eight sources and eight short-distance channels (SDCs). The SDCs were formed by short-distance detectors placed at 8 mm from a given source. The interoptode distance of the standard channels (here on called normal-distance channels, NDCs) ranged from 25 to 40 mm. Sources emitted light at wavelengths 760 and 850 nm, and the light intensity acquired at the detector side was sampled at 7.8125 Hz. The fNIRS cap was placed for each participant according to the measurements taken during the neuronavigation session. Besides the standard cap fixation (using the chin band), the fNIRS was fixated onto the participant’s head with three medical tape stripes (connecting the cap and the participant’s forehead) to assure the cap would not shift during the measurements. In addition, a black, plastic overcap was placed on top of the fNIRS cap to additionally prevent ambient light from reaching the spring-loaded optodes.

#### 2.2.5 fNIRS data analysis

##### Preprocessing

P03 and P21 were excluded from subsequent analysis (see Sec. A.4 in the Supplementary Material for further information). For every subject and run, the raw optical intensity data series were converted into changes in optical density (OD) values using Homer2.^38^ CV values were calculated for the entire run for each channel and those with a CV≥7.5% were discarded from the analysis (see Fig. S7 in the Supplementary Material). Next, the motion detection algorithm *hmrMotionArtifactByChannel* was applied to the OD time-series to identify motion artifacts in each channel. Then, motion-corrected OD data were transformed to change in concentration values through the modified Beer–Lambert law with an age-specific differential path length factor.^39^

##### Assessment of degree of layout (dis)similarity across approaches

The first goal of this study was to assess whether the resulting optode layouts differed across approaches. To do so, the number of overlapping channels and the Euclidian distance between their centers of gravity was computed for each pair of approach-specific layouts, task, and participant. These calculations were averaged across participants afterward and task- and layout-specific frequency maps were computed.

##### Single-run estimates calculation

The short separation regression approach^40^ was applied on the unfiltered Δ[HbO]- and Δ[HbR]-NDC data to remove signal from extracerebral layers of the head. This was done for each NDC and chromophore using the SDC closest to the NDC as the regressor. The SDC-corrected time course was used as input for the *ar_irls* algorithm in NIRS Brain AnalyzIR Toolbox.^41^ This algorithm uses an autoregressive (AR) model for correcting motion and serially correlated errors in fNIRS. The function was adapted to use the ordinary least squares method instead of the *robustfit* approach. The maximum AR model order to be considered was set to four times the sampling rate. The design matrix included the two task predictors convolved with a standard hemodynamic response function (default hemodynamic response function from SPM12). The task predictor for Δ[HbR] was set to −1/3 of the Δ[HbO] amplitude. In addition, a set of low frequency discrete cosine terms were defined as confound predictors using the *dctmtx* function in NIRS Brain AnalyzIR Toolbox with a cut-off frequency of 0.009 Hz.

##### Multirun ROI analysis

We combined the information from both channels comprising each layout to run an ROI analysis as described in Ref. 41 and expanded their procedure to include multiple runs,

$$\beta_{\mathrm{ROI}}=c\beta_{\text{channel}},$$ (1)

$${\mathrm{Cov}}_{\mathrm{ROI}}=c{\mathrm{Cov}}_{\beta }c^{T},$$ (2)

where in this study $\beta_{\text{channel}}$ is the multirun beta estimate and the ${\mathrm{Cov}}_{\beta \mathrm{roi}}$ is the multirun covariance matrix estimated from the concatenated residual time courses and the design matrix. Finally, c is the contrast vector whose coefficients are 0 if the channel does not belong to the ROI and is 0.5 in the two channels that belong to the ROI.

##### Multirun block averages and contrast-to-noise ratio

The SDC-corrected and unfiltered Δ[HbO] and Δ[HbR] time courses were filtered using a zero-phase, band-pass finite impulse response filter of order 1000, with cutoff frequencies of [0.008, 0.25 Hz]. Block averages were computed for each channel and mental-imagery task by taking the average of all trials and runs 4 s before the onset of the task until 15 s after the offset of the task.

The contrast-to-noise ratio (CNR) as was calculated for each channel, ROI and chromophore using the equation described as^35^

$$\frac{\left|\text{mean}(\mathrm{dur})-\text{mean}(\mathrm{pre})\right|}{\sqrt{\mathrm{var}(\mathrm{dur})+\mathrm{var}(\mathrm{pre})}},$$ (3)

where pre represents the rest period from 4 s before onset of task to 0 s; and dur represents the task period from 5 to 15 s post task-onset, as in Ref. 42.

##### Statistical analysis

The second goal of this study was to compare the fNIRS-signal strength and sensitivity obtained from the optodes placed according to the four different approaches. Group differences in terms of CNR and ROI t-estimates were assessed using a nonparametric ANOVA (Friedman test) and follow-up Wilcoxon-paired signed rank tests, one-sided and corrected for multiple comparison with the Benjamini–Hochberg method. Group differences were computed considering: (1) each mental-imagery task separately and (2) all mental-imagery tasks together. In addition, we quantified the number of participants that showed significant increase in the ROI activation.

##### fNIRS data projection onto cortical surface and comparison with fMRI data

We used the inverse distance weighting method described in Ref. 43 and detailed in the Supplementary Material to interpolate fNIRS data on the cortical surface. The projection weights and voxels were used to compute spatially weighted fMRI block averages to assess the temporal correlation between fNIRS and fMRI signals (via Pearson’s correlation). The same computations were performed at both, the single channel and layout level. The latter was used to extract the peak and spatially weighted average t-estimates of individual fMRI activation of the voxels labeled as GM to assess how well the fNIRS ROIs targeted individual activation maps.

## 3 Results

### 3.1 Using Different Information Sources for Optode Placement Results in Different Optode-Layout Designs

**Fig. 6:**
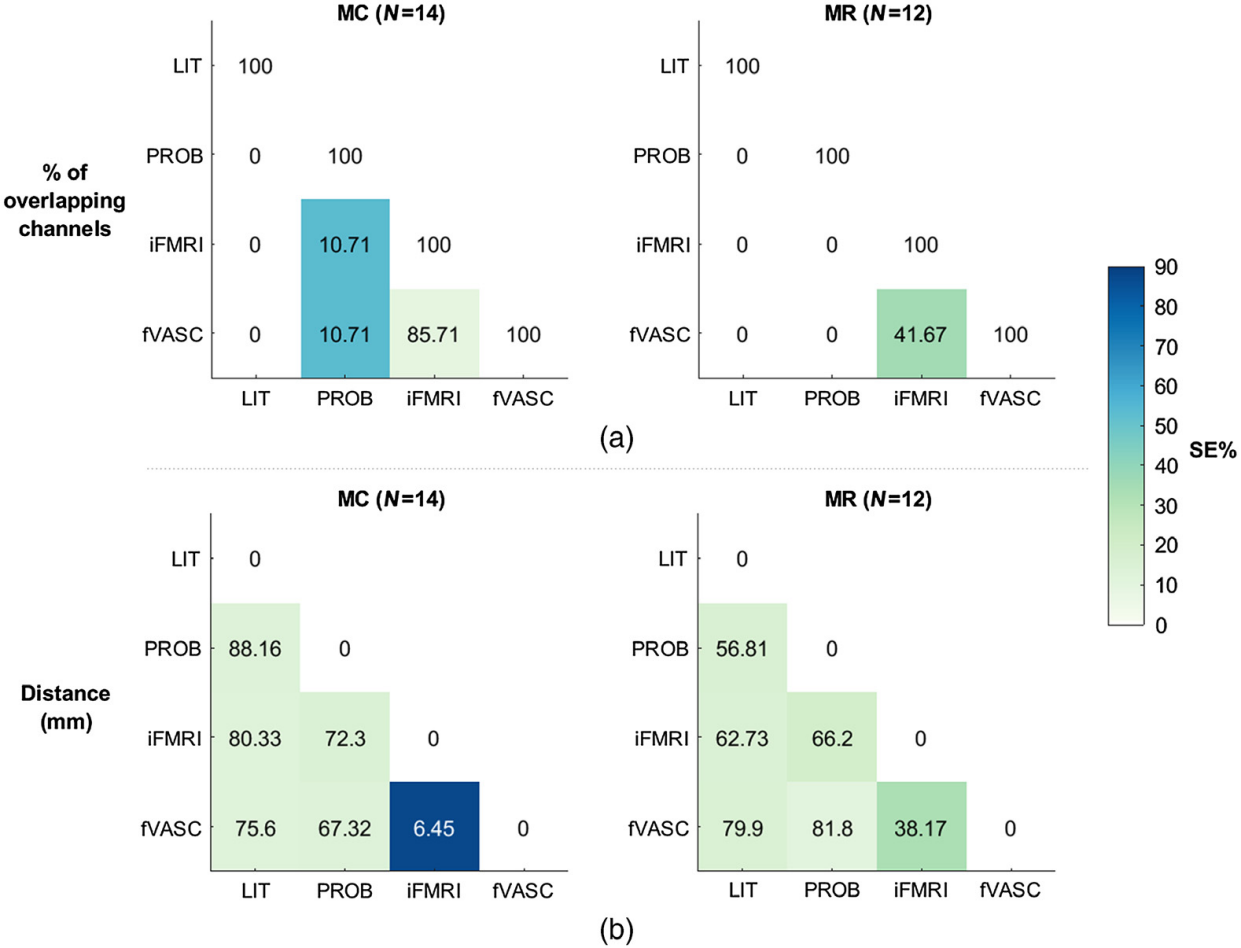
Assessment of degree of layout (dis)similarity across approaches. (a) Average number of overlapping channels for each pair of approach-specific layouts for MC (left) and MR (right) tasks. The numbers in each cell represent (a) the average number of overlapping channels or (b) the average Euclidian distance between COG for each pair of approach-specific layouts for MC (left) and MR (right) tasks. Colors represent the standard error of the mean. MC, mental-calculation; MR, mental-rotation.

Figure 6 shows the mean percent overlap [Fig. 6(a)] and mean Euclidian distance between the COGs of each pair of optode layouts across participants [Fig. 6(b)]. The color of each cell indicates the standard error of the mean. The LIT approach contained no channels that overlapped with the remaining approaches for neither mental-calculation (MC) nor mental-rotation (MR) tasks. Channels placed according to the PROB approach partially overlapped with those from iFMRI and fVASC approaches for MC task. Channels from iFMRI and fVASC approaches overlapped the most. Regarding IS task, P05 showed an overlapping channel between PROB and fVASC layouts (P02 had none). The mean Euclidian distance between the COGs was considerably high (>55 mm) for almost all pair of layouts, which indicates that layouts were located in spatially separated areas. IFMRI and fVASC layouts were located, on average, in close proximity for the MC task (6.45 mm) and to a lesser extent for MR (42.22 mm). The Euclidean distance for IS ranged between 9.08 mm (PROB-fVASC) and 100.19 mm (LIT-iFMRI) for P05 and between 26.83 mm (LIT-PROB) and 75.98 mm (LIT-iFMRI) for P02 (not shown in Fig. 6). Similarly, the frequency maps shown in Fig. S9 in the Supplementary Material indicate that (1) the selected channels vary considerably across participants for PROB, iFMRI, and fVASC approaches; and (2) iFMRI and fVASC show the highest and most similar spatial extension for MC and MR tasks.

### 3.2 Significant Differences in fNIRS Signal Strength and Sensitivity Across the Four Optode-Placement Approaches

**Fig. 7:**
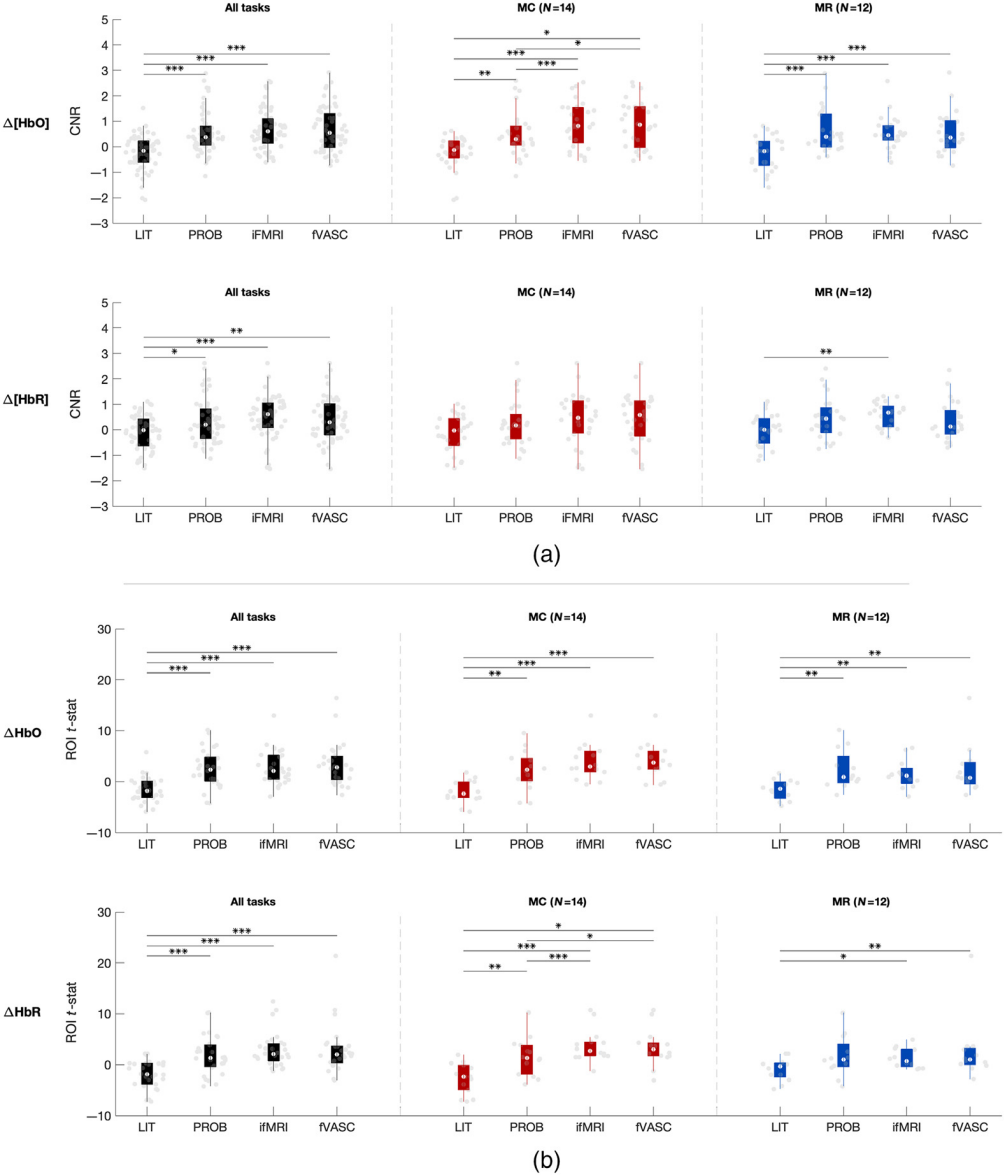
(a) CNR and (b) ROI t-statistics-based group comparison across layouts. Results were evaluated separately for Δ[HbO] (top, in each subplot) and Δ[HbR] (bottom, in each subplot), when all three mental-imagery tasks were considered together as well as separately for MC and MR tasks (left, middle, and right column, respectively). LIT performed significantly worse than the PROB, iFMRI, and fVASC approaches for both chromophores when all tasks were considered together. A similar pattern was observed for MC and MR tasks for Δ[HbO]. Gray dots represent single-subject CNR/ROI t-statistic values for a given mental-imagery task. Whiskers represent the 1.5 times the interquartile range. Significant pairwise differences (calculated using Wilcoxon signed-rank test, one-sided, and corrected for multiple comparisons) are indicated with asterisks: *** = q[FDR]<0.001; ** q[FDR]<0.01; * q[FDR]<0.05. MC, mental-calculation; MR, mental-rotation.

The Friedman test was computed separately for each chromophore (Δ[HbO] and Δ[HbR]) and considering (1) all mental-imagery tasks together and (2) each mental-imagery task separately. For Δ[HbO], CNR and ROI t-statistics significantly differed across layouts (CNR: Fr=41.63, df 4,14, p<0.0001 || ROI t-statistics: Fr=31.66, df 3,14, p<0.0001) when all mental imagery tasks were considered together. Both metrics also also differed significantly across layouts for MC (CNR: Fr=24.67, df 3,14 p<0.0001 || ROI t-statistics: Fr=23.18, df 3,14 p<0.0001) and MR (CNR: Fr=25.72, df 3,12 p<0.0001 || ROI t-statistics: Fr=14.06, df 3,12 p<0.005). *Post-hoc* pairwise comparisons (Wilcoxon signed-rank tests, one-sided) revealed that optodes placed using LIT approach had significantly lower CNR and t-statistics compared to the other three approaches (see Fig. 7). As for Δ[HbR], CNR and ROI t-statistics significantly differed across layouts (CNR: Fr=18.32, df 4,14, p<0.001 || ROI t-statistics: Fr=27.48, df 3,14, p<0.0001) when all mental imagery tasks were considered together. These metrics also differed significantly across layouts for MC (CNR: Fr=7.98, df 3,14, p<0.05 || ROI t-statistics: Fr=15.46, df 3,14, p<0.01) and MR (CNR: Fr=8.23, df 3,12, p<0.05 || ROI t-statistics: Fr=10.56, df 3,12, p<0.05). *Post-hoc* pairwise comparisons showed a similar trend as Δ[HbO]. A similar analysis was carried out based on effect sizes (see Fig. S10 in the Supplementary Material), which showed a similar trend observed in Fig. 7.

Figure S11(a) in the Supplementary Material shows examples of participants with typical hemodynamic responses (a positive deflection in Δ[HbO] and a negative deflection in Δ[HbR]) for the four approach-specific optode layouts, while Fig. S11(b) in the Supplementary Material shows examples of participants with weak/inverted hemodynamic responses for the four approach-specific layouts.

**Fig. 8:**
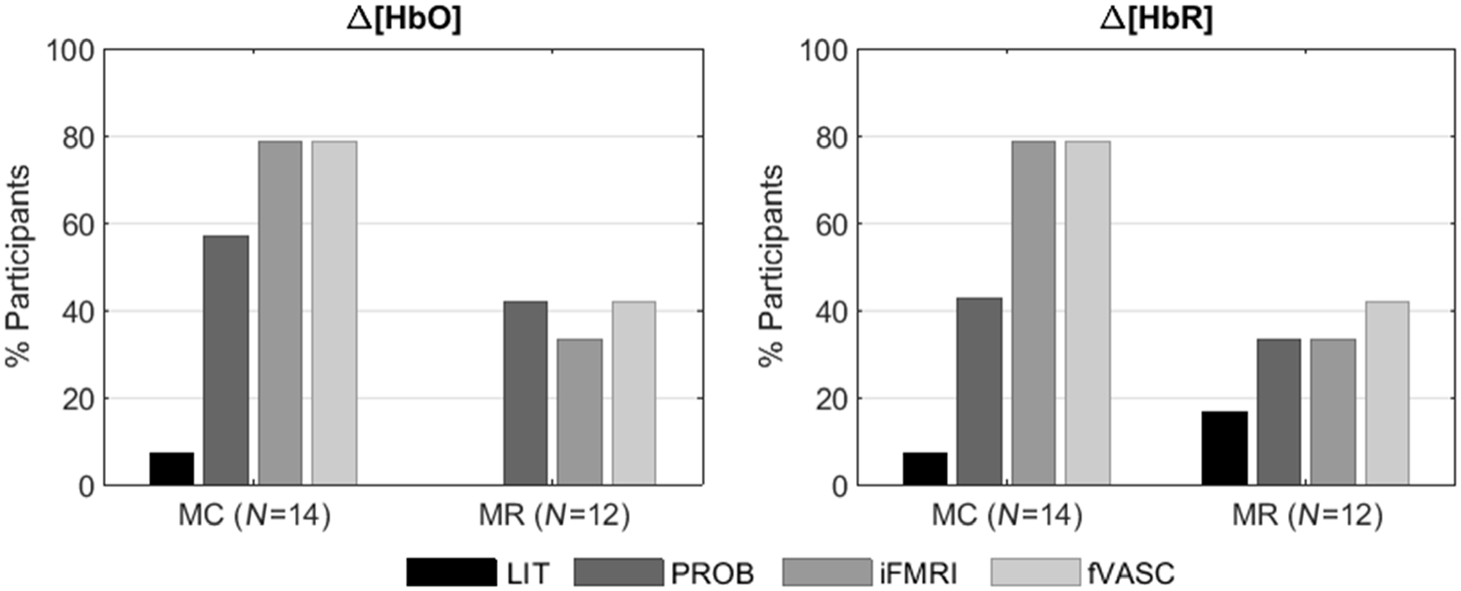
Percent of participants that resulted in significant activation for each mental-imagery task, optode layout, and chromophore. For both chromophores, the percent of participants with significant ROI activation increased with increasing the amount of individualized information used to create optode layouts until a certain point: it plateaued after including individualized functional maps (for MC task) or was slightly reduced after including vascular information (MR task). MC, mental-calculation; MR, mental-rotation.

Figure 8 shows the percent of participants that resulted in significant activation for each mental-imagery task. For both chromophores, the percent of participants with significant ROI activation increased with increasing the amount of individualized information, and plateaued after including individualized functional maps (for MC task) or was slightly reduced after including vascular information (MR task). For the IS task, PROB and fVASC approaches and PROB and iFMRI approaches contained significant ROI activation for both participants (100%) regarding Δ[HbO] and Δ[HbR], respectively. As for MC and MR tasks, the number of participants with significant activation was higher for more individualized approaches than the LIT approach.

### 3.3 Spatial Specificity of fNIRS-ROIs

**Fig. 9:**
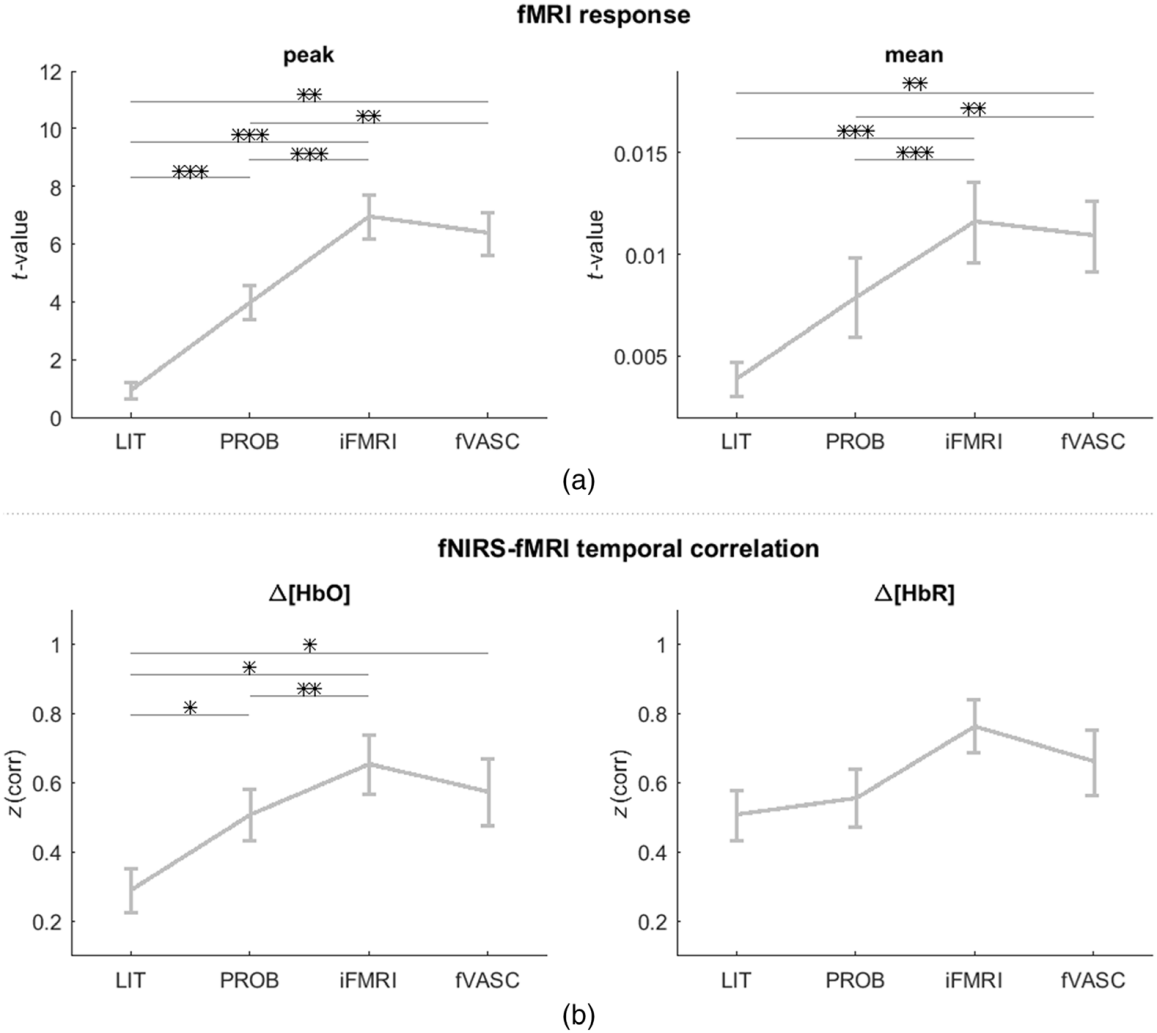
(a) Assessment of layout specificity to fMRI activation maps and (b) of the temporal correlation between fNIRS and fMRI time courses. Peak and average values extracted from fMRI activation maps were highest for channels placed according to iFMRI and fVASC approaches and lowest for the LIT approach, independent of the size of projection spheres used to extract the values (data not shown). Time courses of channels placed according to the LIT approach showed significantly lower temporal correlations with fMRI-signal time courses than following the iFMRI and fVASC approaches. Significance was assessed with Wilcoxon-paired signed tests (one-tailed) and was corrected for multiple comparisons. *** q[FDR]<0.001; ** q[FDR]<0.01; * q[FDR]<0.05.

To assess how well the fNIRS ROIs targeted individual fMRI activation maps, we computed weighted average and peak fMRI responses within the regions of the cortex interrogated by fNIRS channels. The two plots in Fig. 9 show when a sphere with radius r=20  mm was used that both the average and peak responses for LIT are significantly lower than the other approaches (significance assessed by signed rank test and one-sided FDR corrected). Using different sphere sizes did not affect the results (data not shown). The temporal correlation between fNIRS and fMRI time courses [Fig. 9(b)] showed a similar tendency but with smaller differences for Δ[HbO] (and examples of both fNIRS and fMRI time courses are shown in Fig. S11 in the Supplementary Material).

## 4 Discussion

Designing optimized optode layouts is particularly relevant for fNIRS-based BCI and neurofeedback applications, where developing robust systems that use limited number of optodes is crucial to remain practical and comfortable for clinical applications. From the many tools currently available to optimize optode-layout design, we compared four approaches that incrementally incorporated individual information of participants (LIT, PROB, iFMRI, and fVASC) while participants performed mental-imagery tasks typically used in fNIRS-BCI experiments. Our results show that the four approaches resulted in different optode layouts and that the degree of overlap varied across approaches, with the highest overlap and smallest distance between iFMRI and fVASC layouts. Further, channels placed according to the LIT approach showed significantly lower CNR and t-values than those of the channels placed according to the remaining approaches. We observed no significant difference among PROB, iFMRI, and fVASC approaches when all three mental tasks were considered together.

### 4.1 Understanding the Difference in Performance Across Layouts

#### 4.1.1 Lower performance of the LIT approach

**Fig. 10:**
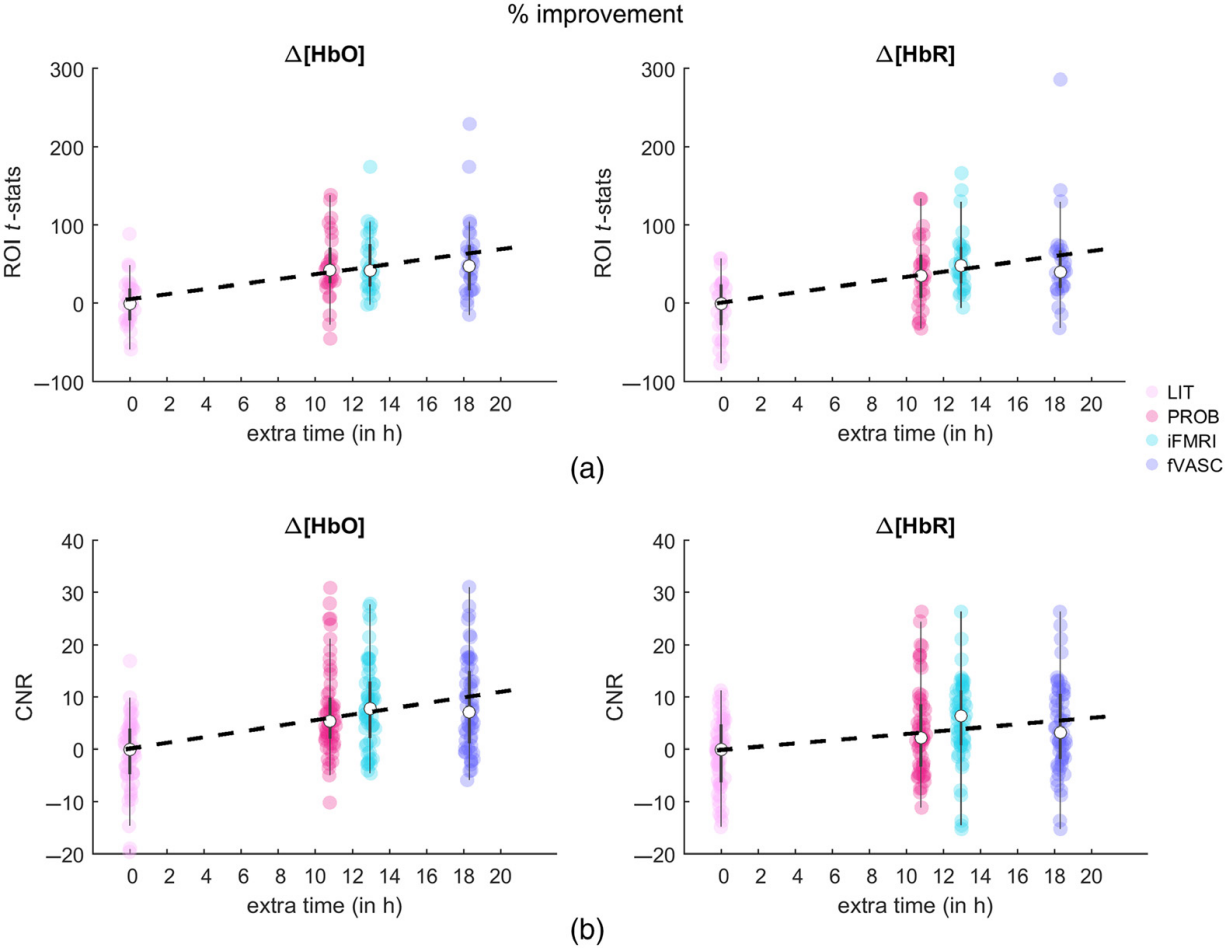
Percent improvement in performance [in terms of (a) t-statistics and (b) CNR)] versus the additional time required to acquire/analyze the data (in hours). All values are relative to the LIT approach (in light pink), here considered the “baseline.” The bigger white circles represent the median of the percent improvement in t-statistics/CNR values for each layout when all three tasks are considered together. The dashed line represents the predicted percent improvement in performance for a given processing time. Points above/below the line indicate that the percent improvement of a given performance measure is higher/lower than the temporal resources spent to achieve that gain.

Figure 10 shows the average and peak responses for LIT were significantly lower than the remaining approaches. The temporal correlation between fNIRS and fMRI time courses showed a similar tendency. These observations were expected since PROB, iFMRI, and fVASC approaches were based on fMRI information. However, if the individual fMRI map is used as the ground-truth measure of cerebral activity due to its superior resolution and higher SNR, Fig. 10 shows that the LIT approach could not capture the underlying signal as good as the other approaches.

Several factors may have contributed to that. First, the head model used for Monte Carlo simulations for LIT differed from the other approaches (Colin27 head atlas versus subject-specific anatomical model, respectively). Although head atlases are good approximations, the tissue geometries may significantly differ from other adults.^44^ Second, mental-imagery instructions used in this study differed from the reviewed studies used for the LIT approach, which may have contributed to a suboptimal selection of the ROIs. Due to the small number of participants for the IS task (N=2), the following lines will focus only on MC and MR tasks. The majority of reviewed papers that reported using (complex) mental arithmetic aimed at increasing the working memory demand and thus mainly measured brain activation in the frontal lobe. In contrast, we asked participants to recite common multiplication tables, which is considered an easy task and thus may have elicited lower responses in frontal and parietal areas when compared to more complex problems.^45^ As for mental rotation, previous work used visually presented cues that had to be mentally rotated, such as geometric objects.^46^^–^^57^ We did not visually present the target object, as participants had to imagine a person rotating while keeping their eyes closed. In turn, unlike the reported studies, there was no reference object to compare to the rotated item. These differences could cause the recruitment of the areas involved in the task to be slightly different.

The agreement between the LIT ROIs and the individual activation maps was assessed by first transforming the former maps to single subject space and subsequently computing the dice coefficient between both maps. For comparability purposes, the same computation was performed between the probabilistic and individual activation maps. A significant difference (p<0.001) between ${\text{dice}}_{\mathrm{LIT},\mathrm{iFMRI}}$ and ${\text{dice}}_{\mathrm{PROB},\mathrm{iFMRI}}$ suggests that the ROIs chosen with the LIT approach are not as predictive of the fMRI maps as probabilistic maps are. In addition, peak t-values for the LIT layout were extracted by masking functional activation maps. The peak t-values were significantly lower for LIT-ROIs than PROB and iFMRI (see Fig. S11 in the Supplementary Material). Overall, these *post-hoc* tests provide some evidence that the ROI definition was suboptimal for the LIT approach.

#### 4.1.2 No significant difference between fVASC and iFMRI layouts

The fVASC and the iFMRI approaches only differed in the number of tissues used during Monte Carlo simulations: the fVASC condition included additional participant-specific vascular information. Including additional vascular information did not result in a significant difference compared to the iFMRI layout at the group level. This is mainly because the generated layouts were similar between them, as indicated by the channel overlap across layouts and the Euclidian distance (Fig. 6). This high similarity seems to be driven by the functional ROIs, which was the same for both approaches. Our decision to use a small number of optodes for each layout, the constraints to select them, and segmentation-related factors (see the limitations Sec. 4.2.2) may have also limited the improvements expected from the fVASC approach.

#### 4.1.3 PROB performs similar to iFMRI and fVASC

We observed that CNR and t-statistics performed similarly for the PROB approach compared to the iFMRI and fVASC approaches. Figure 9 also shows that, descriptively speaking, the peak and average values captured by channels defined based on the PROB approach are closer to those of iFMRI and fVASC approaches than the LIT approach is. This is because PROB approach-based activation maps show high spatial correspondence when compared to the reference fMRI maps for each participant and mental task. Indeed, the average spatial correlation (assessed by Spearman correlation) between probabilistic maps and individual activation was 0.63 when all tasks are considered together and of 0.63 for MC and 0.64 for MR tasks. For IS, the values ranged between 0.52 and 0.66. These values, together with the results presented in this study, suggest that using probabilistic maps defined on individual anatomical space can be used for a new subject (as long as the functional maps used to create the probabilistic maps are based on the same or similar task).

### 4.2 Optode-Layout Design and Its Limitations

#### 4.2.1 Cost function, optimization problem, and constraints

The cost function maximized the total sensitivity to the preselected ROI and was the same as in Ref. 5. However, the algorithmic approach to solve the optimization problem was tailored to account for the constraints imposed by our particular research question(s), experimental design, and the nature of our ROIs (which consisted of multiple noncontiguous regions). This entails that our algorithmic approach may not be (and was not designed to be) generalizable to other experimental designs.

As for the optimization constraint set, the optode layout for each of the four approaches consisted of two channels that shared one optode. This was motivated by its suitability for clinical settings. However, due to recent technological developments, wearable, ergonomic fNIRS instruments^58^ with a high number of optical channels and easy setup are becoming a reality,^1^ which could relieve this optimization constraint in the future.

#### 4.2.2 Monte Carlo simulations

Our light-sensitivity profiles may contain estimation errors due to a number of simplifications. First, the head models used in this study did not consider that the skull can contain cancellous and cortical bone, and the soft tissue may contain fat and muscle that have different optical properties.^59^ Second, both sources and detectors were modeled as pencil sources instead of separately being modeled according to their function (they emit or detect light) and technical characteristics. Third, we did not distinguish between arteries and veins when defining the head model. Even if our decision can be justified by the relatively small difference in optical properties between veins and arteries compared to the remaining tissues, we cannot discard divergence in the results if arteries and veins had been distinguished. Optical properties also differ depending on the diameter of blood vessels,^60^ which we did not take into account in this study. Finally, our vascular maps depended on manual segmentation, which may have introduced variability. Future studies may overcome these limitations by mapping superficial (scalp/skull) vasculature with more optimized MRI sequences,^61^ and by distinguishing between arteries from veins and their diameters.^62^^,^^63^

#### 4.2.3 Mental-imagery task selection

Combining approach-specific layouts for both mental-imagery tasks caused incompatible optode placements in some participants. The decisions taken to overcome these problems, together with the subject-specific task selection led to an unequal number of participants for each task ($N_{\mathrm{IS}}=2$, $N_{\mathrm{MC}}=14$, $N_{\mathrm{MR}}=12$), which made the group analysis for IS task unfeasible. To overcome the incompatibility problem, future studies could test the performance of different layouts in different runs/sessions (using a given layout at a time), whose order could be counterbalanced to account for run/session effects. In addition, a single mental-imagery task could be studied at a time.

### 4.3 Implications for BCI Applications

In fNIRS-BCI applications for motor-independent communication and control, brain responses from a set of tasks are discriminated by exploiting information in distributed patterns of brain activity using multichannel pattern analysis (the equivalent to multivoxel pattern analysis in fMRI studies) or using univariate analysis in combination with smart paradigms.^10^^–^^12^^,^^16^^,^^64^^,^^65^ For either approach, it is important to ensure there is a set of channels that contains sufficient task-related information to discriminate responses. This study constitutes a relevant prestep for both as it compared approaches that used different amount of individualized information to design task-specific, optimized layouts that should result in informative channels.

The percent of participants with significant activation increased with the amount of individualized information used to create the optode setup, but only until a certain point (adding vasculature information did not increase or even reduced the percent of participants for MC and MR, respectively). Although all participants showed significant activation levels for every mental task during the fMRI run, none of the approaches using fMRI information managed to get all participants to have significant ROIs for MC and MR tasks. It is unclear whether a given level of fMRI activation is enough to guarantee the detection of task-related fNIRS signal. Even if both neuroimaging methods measure the hemodynamic response to neural activity, fNIRS is highly dependent on the individual anatomical features, such as the scalp-brain distance (which differs across the head).^17^ In addition, our fNIRS results might have been affected by the discrete spatial locations used in this study (130 EEG positions). Spatially less restricted or unrestricted optode placement would likely improve the results.^66^

### 4.4 Recommendations for Optode Placement and the Way Forward

Effective optode-layout design balances a number of potential tradeoffs. The extended layouts based on the international 10-20 system or its extensions can be used to study functional network dynamics and are adequate when target ROIs are not easy to define.^66^ In addition, although the target ROI may not be optimally sampled (due to unavoidable regions not covered by a source–detector pair when creating optode layouts and the lower spatial resolution associated to fNIRS compared to fMRI), the chance of completely missing it is relatively low. That said, smaller setups are preferred in fNIRS-BCI applications due to their superior practicability and patient comfort. However, they run a higher risk of missing signal from the target ROI due to anatomical or functional differences between individuals. As a result, small BCI setups are likely to benefit from supplementary f/MRI data investigated in this work. The recommendations and conclusions presented here therefore focus on this particular fNIRS application.

Considering that additional individualized information has an associated acquisition/analysis cost, it is worth asking, especially when temporal/monetary/material resources are limited: how much individual information is worth to include for designing optode layouts? Figure 10 shows the predicted percent improvement in performance (in terms of t-statistics [top] and CNR [bottom]) versus the additional time required to acquire/analyze the data relative to the LIT layout (considered the “baseline” approach). Points above the line indicate that the percent improvement of a given performance measure is higher than the temporal resources spent to achieve that gain. The figure suggests that including individual anatomical data with independent (PROB layout) or individual functional data (iFMRI layout) improves the performance while efficiently using temporal resources. It also suggests that the fVASC approach in its current form is not as cost-effective.

The analysis described above focused only on a small part of the multidimensional problem related to cost-effectiveness. Naturally, costs and benefits of including more individualized information for creating clinically practical layouts should be assessed in that very context. For example, in certain (rare) cases such as long-term BCIs in “locked-in” patients, using individual (f)MRI data may result in increased ability to communicate, i.e., provide considerable benefit. In that case, even though using individual (f)MRI is more resource-demanding, the benefits would outweigh the costs.

In view of these observations, we encourage researchers to use individual functional and anatomical data for designing optode layouts when possible, but when anatomical data are available and functional data are not, probabilistic functional maps constitute a promising and economic alternative. FMRI-based probabilistic functional maps of the human ventral occipital cortex,^67^ human motion complex,^68^ face selective areas,^69^^,^^70^ finger dominance in the primary somatosensory cortex,^71^ or across the whole cortex^72^ are freely available or available on demand. However, we could not find any published work on probabilistic mental-imagery maps, which could be beneficial for optode placement in BCI research. To improve this situation, the probabilistic functional maps of the three mental-imagery tasks used in this study (in MNI space) are available upon request. Finally, in the absence of functional and anatomical information (for example, when a patient cannot go into the scanner for incompatibility or cost-related reasons), a few strategies could be employed. One of them involves using functional probabilistic maps in combination with atlas-based anatomical information. Even though this approach was not tested in this study, we believe it can be beneficial. If probabilistic maps are unavailable, ROI selection should be guided by relevant body of work or meta-analyses that describe tasks closely related to the ones to be used during the fNIRS session. In parallel, a larger setup could be initially employed in a “localizer” run to determine the most informative channels which could be subsequently scaled down to consider only the most informative channels. In this study, once the target ROIs were selected, we used fOLD^29^ for designing our optode layout due to its user-friendly features. However, other toolboxes such as Array Designer^1^ and software, such as NIRStorm (a *BrainStorm* plugin for fNIRS analysis^27^), also offer promising and flexible tools that were not explored in this study.

## 5 Conclusions

In this paper, we compared four approaches to design small fNIRS optode layouts that represent various scenarios research groups may encounter when planning fNIRS-BCI experiments. By providing the insights of such comparisons, we hope to have offered an informative framework so that researchers can efficiently use resources for developing robust and convenient fNIRS-BCI systems for clinical use.

## Code, Data, and Materials Availability

Code supporting the analysis and results can be found in https://github.com/AmaiaBA/Neurophotonics-2021; raw data will be available upon request to the authors.
