# Supplementary material for: Guiding functional near-infrared spectroscopy optode-layout design using individual (f)MRI data: effects on signal strength
Source: Neurophotonics. 2021 Jun 17;8(2):025012. doi: 10.1117/1.NPh.8.2.025012 (PMC8211086; doi:10.1117/1.NPh.8.2.025012)
Supplement: Supplementary file 1 [file NPh_008_025012_SD001.pdf]

A. Materials and Methods

Participants

Some of the initially recruited twenty-one participants for the f/MRI session became unavailable over the session (see Table S1).

Table S1. Summary of participants' characteristics and involvement of different experimental sessions.

Participant ID	f/MRI (N=21)	Neuronavigation (N=17)	fNIRS (N=16*)	Gender	Age range	Handedness
**P01**	YES	YES	YES	Female	25-30	Left
**P02**	YES	YES	YES	Female	25-30	Right
**P03**	YES	YES	YES*	Male	25-30	Left
**P04**	YES	YES	YES	Female	25-30	Right
**P05**	YES	YES	YES	Female	25-30	Right
**P06**	YES	YES	YES	Female	40-45	Right
**P07**	YES	**NO**	**NO**	Male	25-30	Right
**P08**	YES	**NO**	**NO**	Male	30-35	Right
**P09**	YES	YES	YES	Male	25-30	Right
**P10**	YES	YES	YES	Female	25-30	Left
**P11**	YES	YES	YES	Female	25-30	Right
**P12**	YES	YES	**NO**	Female	25-30	Right
**P13**	YES	**NO**	**NO**	Male	20-25	Right
**P14**	YES	YES	YES	Male	30-35	Right
**P15**	YES	YES	YES	Male	30-35	Right
**P16**	YES	YES	YES	Male	25-30	Right
**P17**	YES	YES	YES	Female	20-25	Right
**P18**	YES	**NO**	**NO**	Male	25-30	Right
**P19**	YES	YES	YES	Female	20-25	Left
**P20**	YES	YES	YES	Female	20-25	Right
**P21**	YES	YES	YES*	Female	25-30	Right

Note: P03 and P21 were excluded from fNIRS analyses, see section A.3

A.1 Acquisition of structural, functional and vascular MRI data

We used a magnetization prepared-rapid gradient echo (MPRAGE) sequence to collect structural T1-weighted MRI data, with the following parameters: repetition time (TR)=2250ms, echo time (TE)=2.21ms, inversion time (TI)=900ms, flip angle (FA)=9°, number of slices=192, 1-mm isotropic resolution, duration=5:05min. 2D Gradient Echo echo-planar imaging sequence with a TR=1s, number of slices=36, and 3-mm isotropic resolution was used to acquire functional data. Cerebral and pial vascular data was collected using 2D- and 3D- Time-of-Flight (TOF) sequences (FA=60º/18º, TR=21ms/20ms, TE=4.83/3.3ms, number of slabs=1/5, number of slices in slab=75/40, with distance factor=-33/-20%, 0.7-mm isotropic resolution, duration=9:11/4:56min). Finally, scalp-vascular data was obtained with a Multi-Echo Gradient Echo (GE) sequence with four different echoes (TR=34ms,

$TE_1/TE_2/TE_3/TE_4$=3.02/8.56/15.11/23.91ms, number of slices=192, 0.7-mm isotropic resolution, duration= 8:06min).

Training session prior to the functional run

During training, participants had to recite overtly the chosen text and the multiplication tables for the inner-speech and mental-calculation tasks, respectively to ensure the speed was consistent, and to repeat the same procedure covertly until they felt comfortable with the tasks. As for the mental-rotation task, participants watched short clips of a jumping diver/gymnast until they could comfortably imagine the movement.

Examples of participants' strategies

Table S2. Example of participants' strategies

IS	*"I recited the national anthem. I started from the beginning with each trial. I imagined hearing myself and really put emphasis on certain words, as if I was reciting in front on an audience"*<br><br>*"I recited the 'mantra' Our Father in my mother tongue, I did it sometimes as fast as possible sometimes on normal speed"*<br><br>*"I recited the poem 'we wear the mask' by Paul Dunbar"*
MC	*"I recited tables of 7,8,9 intermixed"*<br><br>*"I switched between multiples of 7 and 8 to keep the difficulty level about the same over trials. Always started from the beginning for each trial"*
MR	*"I imagined a floor gymnast performing back flips and ending the length with a triple rotation. This continued with back flips in the other direction"*<br><br>*"I imagined a diver in mid-air rotating with his legs stretched. Infinitely long fall"*<br><br>*"I imagined people diving into the pool and rotating a lot"*

A.2 Analysis of structural, functional and vascular MRI data

Functional data

Preprocessing

Data were pre-processed using inter-scan slice-time correction, 3D rigid-body motion correction (applying Trilinear interpolation for detection/sinc interpolation, for correction), and temporal high-pass filtering with a general linear model (GLM) Fourier basis set of 3 cycles/run. Functional data of

3-mm iso-voxel resolution were spatially co-registered to the structural image by using a gradient-based intensity-driven fine-tuning alignment.

fNIRS coverage mask definition

Deeper voxels that were active in the individual functional maps were masked by the fNIRS coverage mask. A whole-head mask was created for each participant from the bias-corrected structural image. Each mask was iteratively eroded 50 times and all voxels that did not belong to this eroded mask were selected and intersected with the original head mask. The 'surviving' voxels were used to mask out active voxels from deeper regions, as we did not expect the fNIRS signal to be sensitive to these regions [44].

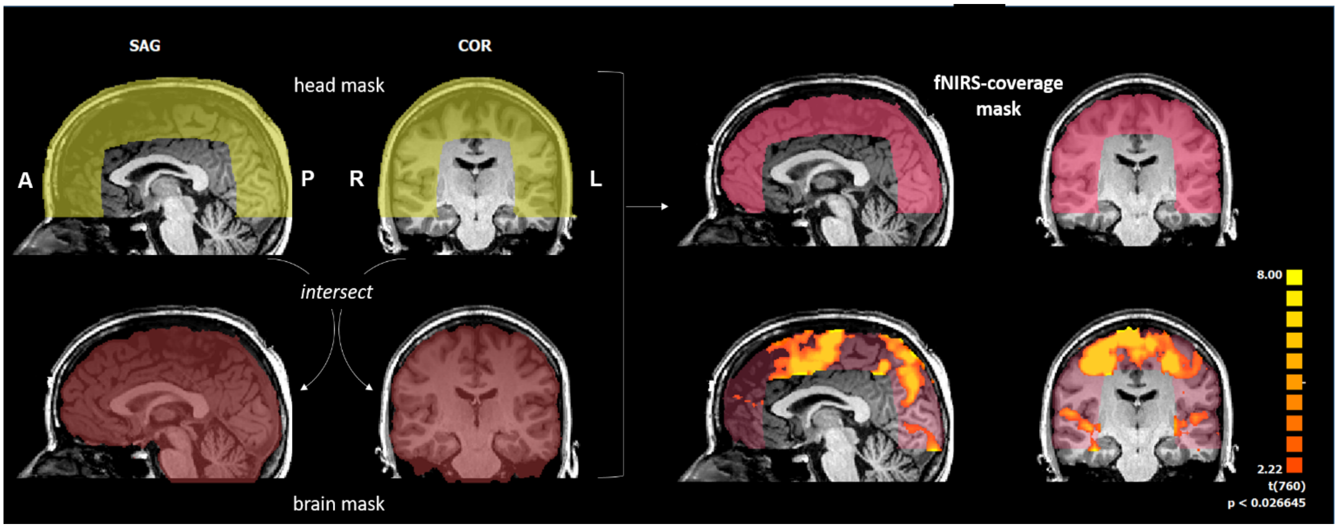

Fig. S1. fNIRS-coverage mask definition and application. An fNIRS-coverage mask was created by the intersection of the eroded head mask and the brain mask, and it was used to mask out the active voxels from deeper regions. The activation map depicted in this figure resulted from the MR vs. Rest contrast for a representative participant. The activation map was corrected using a cluster-extent threshold at 5%.

Probabilistic functional maps

While it is not uncommon for researchers to have previously acquired anatomical MRI data of the same participant [73, 74] (which may be available as part of the medical record or a research database), having individual anatomical *and* functional data of the same participant represents a less likely scenario [74]. In the absence of individual functional data, probabilistic functional maps can be generated from other individuals whose functional data are available.

We defined subject-specific probabilistic functional maps based on an independent sample, i.e., the functional surface activation maps from the remaining individuals, that had been aligned to a common space. Cortex-based alignment (CBA) is a whole-cortex alignment scheme [72, 75-77] which uses curvature information of the cortical surface to iteratively reduce misalignment across participants and in turn increase functional overlap on the group level [74]. For that, individual WM reconstructions of each

hemisphere were aligned to a dynamically generated group average (N=21) and the resulting individual transformation files were used to align the individual activation maps.

The MR *vs.* Rest map from P08 was excluded from subsequent analyses as the participant reported not being able to perform the mental-imagery task correctly and having used an alternative cognitive strategy instead. Thus, the maps for each participant were created based on N=20 participants for the IS and MC tasks and based on N=19 participants for the MR task. We discarded mesh vertices that were active in less than 20% of the sample size for each task and hemisphere. The resulting maps for each hemisphere were transformed back into individual volume space by interpolating from -1mm to +3mm from the GM/WM segmentation boundary and smoothed with a 2mm full-width-half-maximum kernel.
Fig. S2 depicts an example of probabilistic functional maps for each of the mental-imagery task, from a left, top and right view. Colors represent the percent overlap of significant activation across participants for a task vs. rest contrast (corrected with a cluster threshold that allowed for a 5% loss of active voxels).

Vascular data

Cerebral and Pial vasculature

2D and 3D TOF data were aligned individually to an up-sampled version (0.7-mm isotropic resolution) of the anatomical data of the same session for each participant, following the same co-registration approach as for functional maps described above. Vascular data were segmented with automatic segmentation tools in BrainVoyager QX (intensity-based segmentation) and the software *Segmentator* (intensity gradient-based segmentation [78]) and manually corrected when necessary. The latter was done using ITK-snap [79] and BrainVoyager QX. The segmented vascular structures from 2D and 3D TOF data were then combined and were down-sampled to 1-mm isotropic resolution. The analyses procedures are summarized in a flow-chart diagram (Fig. S3) and an example reconstruction is shown in Fig. S4.

Scalp vasculature

All four echo images derived from the multi-echo GE protocol were first aligned to the 0.7-mm isotropic resolution anatomical images for each participant. We then isolated the extracerebral tissues by masking out the brain using *FSL BET* v5.0 [80]. Depending on which image(s) showed higher contrast for vascular structures, segmentation was performed manually in BrainVoyager QX using a combination of the four echoes or using the later echo images, *i.e.*, TE_3=15ms and TE_4=23ms, which showed higher contrast for vascular structures than earlier echoes. The segmented vascular structures were then down-sampled to 1-mm isotropic resolution. The analyses procedures are also summarized in the flow-chart diagram provided in Fig. S3 and an example reconstruction is shown in Fig. S4.

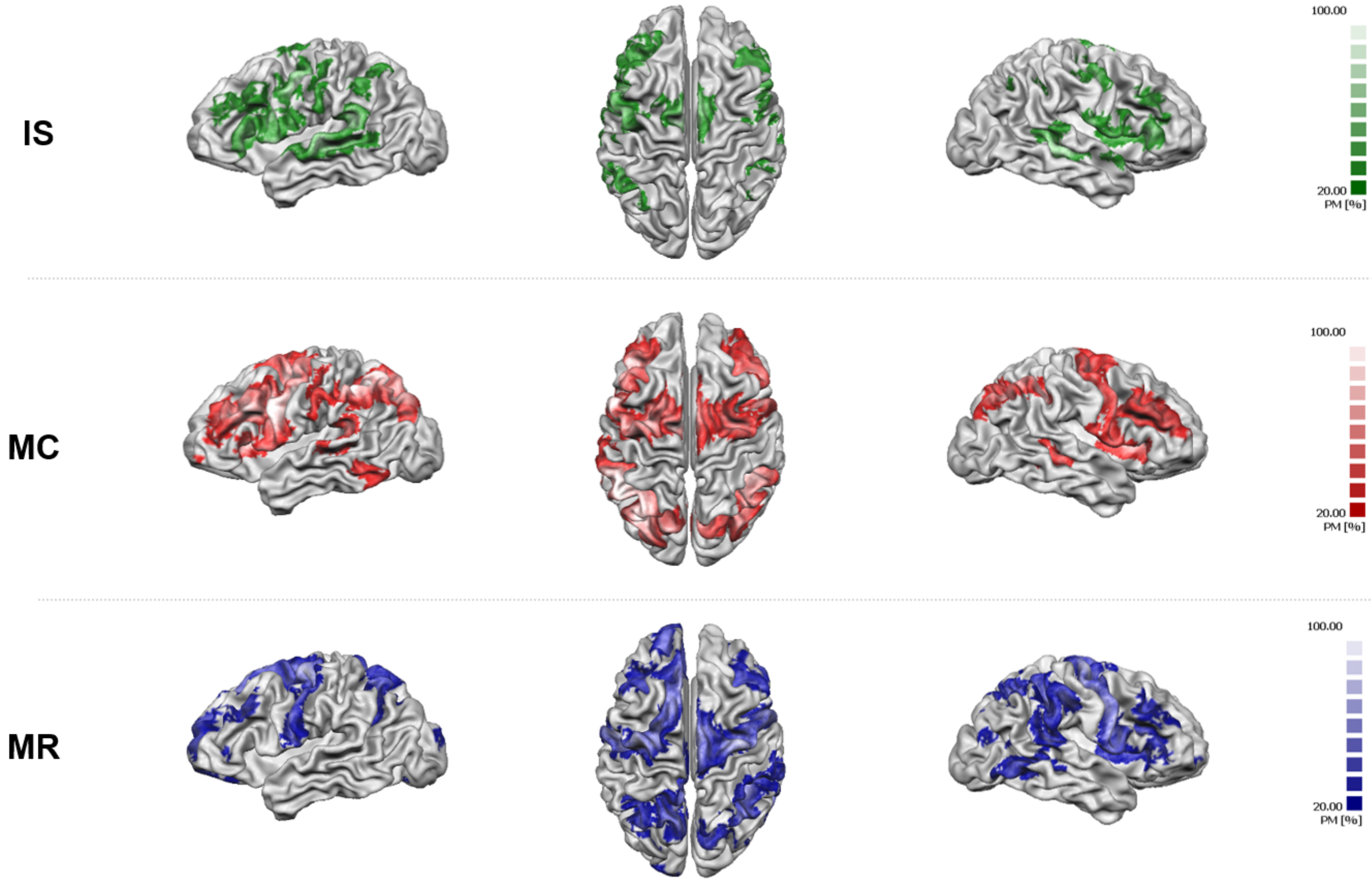

Fig. S2. Example of functional probabilistic maps (left, top and right view) for each mental-imagery task (IS= inner speech; MC = mental calculation; MR = mental rotation).

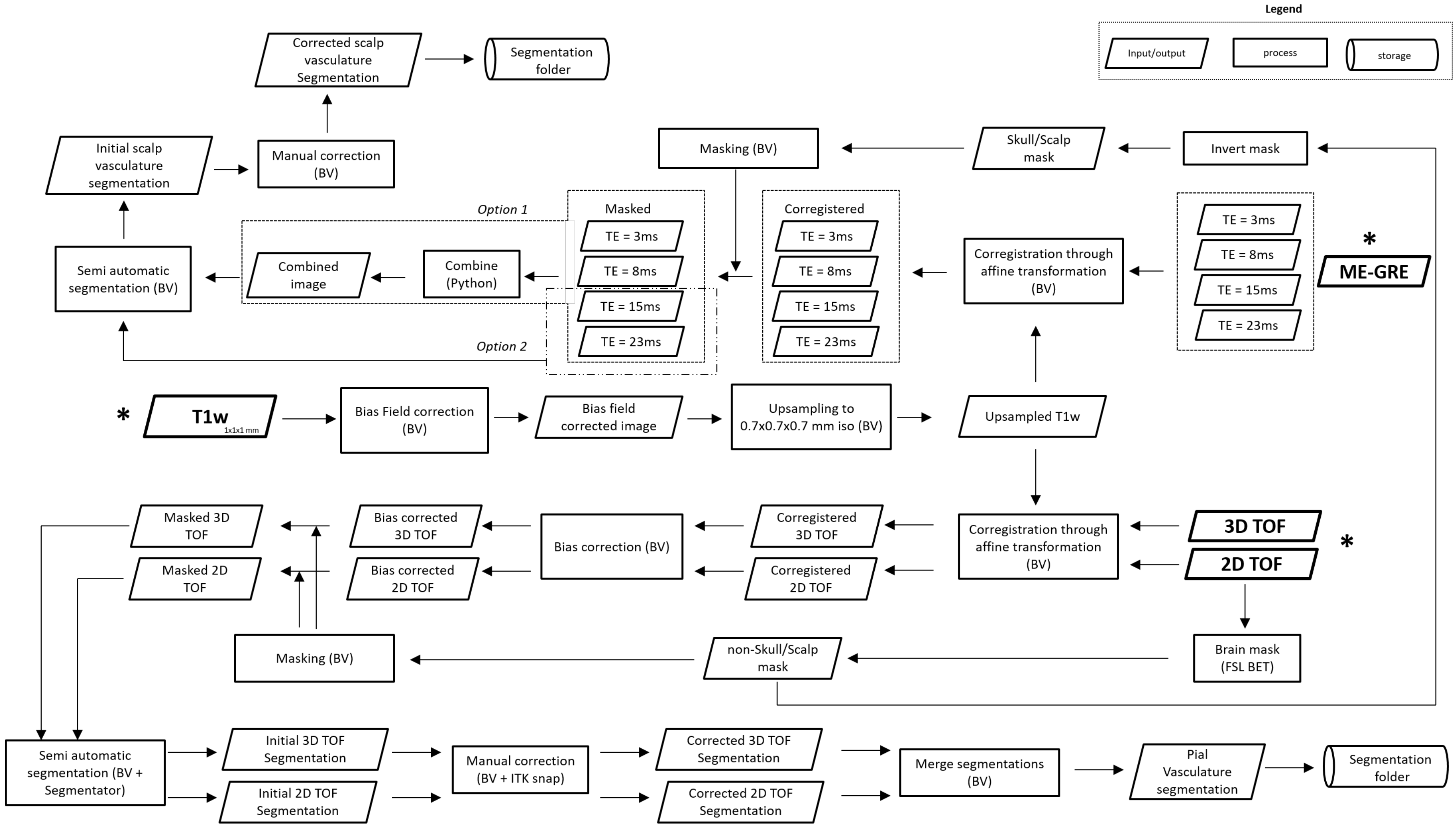

Fig. S3. Steps involved in vascular data segmentation for each participant. Asterisks indicate starting points in the pipeline. BV = Brainvoyager QX; TOF = time-of-flight; T1w = structural (MPRAGE) image; ME-GRE: multi-echo gradient echo images.

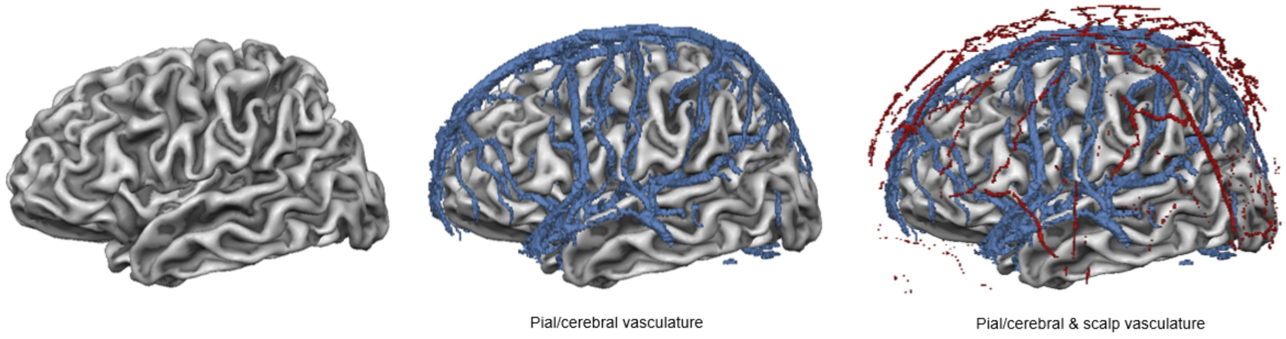

Fig. S4. Example of vascular reconstructions (P16). Left. Cortical reconstruction (left hemisphere). Middle. Pial/cerebral vasculature reconstruction (in blue) overlaid onto the cortical mesh. **Right**. Scalp vasculature reconstruction (in red) overlaid onto the cortical mesh and the pial/cerebral vasculature reconstruction.

A.3 Neuronavigation session

To ensure consistent cap placement between the Neuronavigation and the fNIRS sessions, a careful approach was followed. First, the head circumference for each participant was measured using a measuring tape. The cap was placed on and was secured using a chin band. Next, its position was adjusted so that the Cz location would be exactly half the nasion-inion distance. The inion was defined as the top part of the pronounced structure in the occipital region. In order to ensure that the cap was not tilted or shifted to one side, the distance between the left and right pre-auricular points was measured and the cap was gently moved in this virtual coronal plane until Cz was located half this distance. The preauricular points were defined as the location where the mandibular bone moves with the opening and closing of the mouth. Finally, the cap was secured with medical tape on the forehead to prevent any unwanted cap shift. The Cz location details (in terms of nasion-inion and pre-auricular distance) together with the cap size were noted down for the fNIRS session.

Single ultrasound markers (three in total) were attached to the participant's head using adhesive stickers. Next, three reference points (inion and left and right preauricular points) defined on the participant's head were used for the co-registration of the structural MRI image with the participant's head in the external (real) world. Once these steps were completed, the 130 EEG locations marked on the cap were digitized.

A.4 Preparing for fNIRS signal acquisition: optode layout creation

<u>Monte Carlo simulations</u>

The way light sensitivity profiles were computed across four approaches differed in a number of aspects besides the software used (see Table 2). The following section summarizes those differences:

1. <u>Head models and tissue segmentations</u>

Monte Carlo simulations require the anatomical head models to be segmented into different tissues. This is necessary for photon-transport simulations as different tissues of the human head present different optical properties (absorption, scattering, anisotropy and refraction). For the LIT approach,

we used the MNI Colin27 head atlas (the default atlas available in FOLD). FOLD uses a five-layer segmentation of the MNI Colin27, which consists of scalp, skull, CSF, GM and WM tissues. For the remaining approaches, a five-layered model was created from the individual anatomical images using a hybrid segmentation algorithm [73]. This algorithm, developed in MATLAB and available upon request from the authors, takes as input the standard GM and WM segmentations of a T1-weighted image from FreeSurfer and applies sequential morphological operations implemented in iso2mesh tools to accurately reconstruct skull, scalp, and CSF layer thickness. The GM and WM segmentation images were created in FreeSurfer v06 [81]-using the standard processing stream (recon –all, which took ~10 h per participant). The resulting tissues from the hybrid segmentation algorithm were converted into compatible BrainVoyager QX files to visually inspect and manually correct them if necessary. Although GM and WM segmentation files had been created in BrainVoyager QX in a previous step (see Sec. 2.1.3), the automatic segmentation in BrainVoyager usually disregards the cerebellum. We thus used the segmentations from FreeSurfer to create a head model for Monte Carlo simulations. From the corrected segmentation files, a single image file was created by assigning integer values ranging from 1 to 5 to the different tissues (as in FOLD). Specifically, voxels corresponding to scalp were assigned the value 1, voxels corresponding to skull were assigned value 2, CSF 3, GM 4 and WM 5. The remaining voxels were assigned value 0 (air). We ensured that voxels inside the head were not assigned the value 0 by first identifying them and subsequently assigning the value dictated by their direct neighbors. The fVASC approach differed from the PROB-based and the iFMRI-based approaches in that vascular structures were included in the head model. For that, both pial/brain and scalp vasculature segmentations were combined and included as the sixth layer. To prevent voxels being assigned to two different tissues simultaneously, all voxels considered as vascular tissue were removed from the remaining five tissues. Importantly, our segmentations could not distinguish veins from arteries and all voxels were treated as veins. Both, five- and six-layered models are shown in Fig. S5.

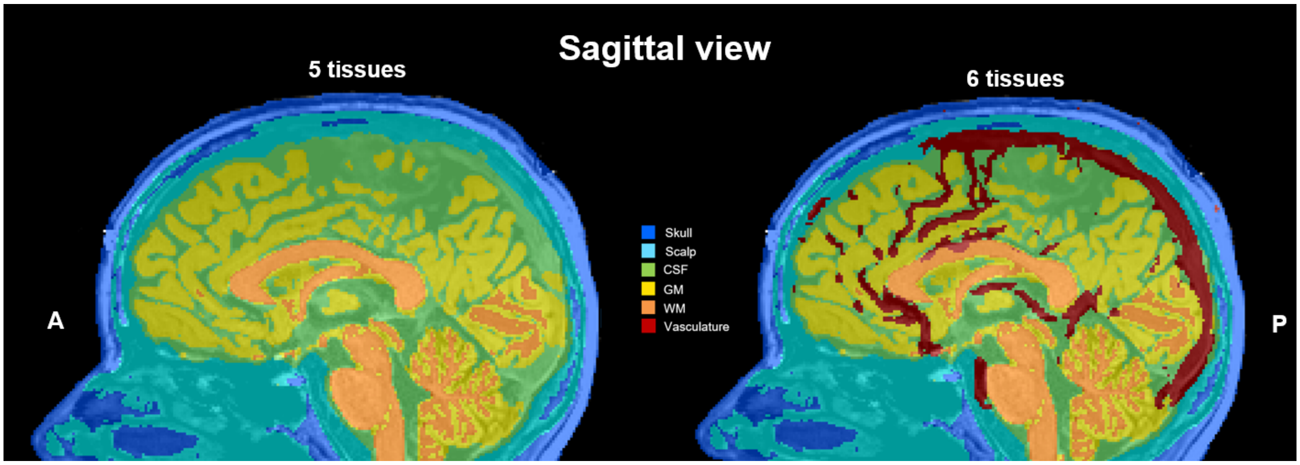

Fig. S5. Example head model (P01) used for Monte Carlo simulations. Left. Five-tissue model used for PROB and iFMRI. **Right.** Six-tissue model used for fVASC.

2. Optical properties

For comparability purposes across approaches, we used the average optical properties across four NIRS wavelengths (690, 750, 780 and 830nm) as in FOLD. We defined the optical properties of vascular structures based on the scattering, absorption and anisotropy values provided by [30]. Specifically,

the oxygen saturation of blood (SO_2) is defined as the ratio of the HbO_2 concentration to the total hemoglobin concentration, where SO_2[Arteries] ~ 97.5% and SO_2[Veins] ~ 75%. Bosschaart, Edelman [30 reported optical coefficients for a range of wavelengths and SO_2 of 98% and SO_2 of 0%. SO_2=98% can be considered an approximation of the arterial blood and a combination of both (SO_2=98% and SO_2=0%) can be used to approximate the venous blood:

$$SO_2[Veins] = 0.75 \times SO_2[98\%] + 0.25 \times SO_2[0\%] \quad (1)$$

Table S3. Optical values reported in Bosschaart et al. (2014) for the four wavelengths used in the present study. The scattering (µs) and anisotropy (g) parameters were based on a mathematical model (proposed model, column 1 in the present table), while the absorption parameters (µa) based on empirical data (column 2). The columns in *italics* correspond to optical values for venous blood which was calculated based on a weighted sum of SO2 98% and 0% values (equation 1).

	proposed model						empirical data		
λ (nm)	µs (mm^{-1}) SO2 (98%)	µs (mm^{-1}) SO2 (0%)	*µs SO2 (75%)*	g SO2 (98%)	g SO2 (0%)	*g SO2 (75%)*	µa (mm^{-1}) SO2 (98%)	µa (mm^{-1}) SO2 (0%)	*µa SO2 (75%)*
690	85,84	75,63	*83,2875*	0,9843	0,9852	*0,9845*	0,13	1,17	*0,39*
750	77,04	67,62	*74,685*	0,9827	0,9836	*0,9829*	0,24	0,81	*0,3825*
780	72,59	63,73	*70,375*	0,9819	0,9827	*0,9821*	0,33	0,59	*0,395*
830	66,96	58,72	*64,9*	0,9804	0,9812	*0,9806*	0,46	0,43	*0,4525*

Table S4. Optical properties of arterial and venous blood based on the optical values reported in Bosschaart et al. (2014). Values were computed as the average of four wavelengths used in the present study.

	µa (mm^{-1})	µs (mm^{-1})	g
**Arterial blood (SO2 98%)**	0,29	75,6075	0,982325
**Venous blood (SO2 75%)**	0,405	73,311875	0,982525

3. ROI selection and definition

The ROIs for the LIT approach were selected based on a literature review of the three mental-imagery tasks used in this study. We used the PubMed database and keywords for the search included: 'inner speech', 'covert speech', 'mental talking' and 'overt speech' for inner speech (overt speech was also included as both covert and overt speech share common networks[82]),'mental calculation' and 'mental arithmetic' for mental calculation; and 'mental rotation' for mental rotation. We included every work that reported using such mental-imagery tasks, independent of the neuroimaging modality and the population under study (see Table S5). These ROIs were then defined in the MNI Colin27 brain based on the Jülich histological atlas available in FOLD. ROIs for each mental-imagery task were selected based on the most-frequent regions reported across studies. This number different across the three tasks (see Table S6). The selected ROIs for the PROB-based approach were the active regions of the

individual probabilistic mental-imagery maps. For iFMRI and the fVASC approaches, individual mental-imagery contrast maps were used as ROIs (see Sec. 2.1.3).

4. Inter-optode distance

FOLD performs the Monte Carlo simulations on neighboring optical positions of 10-10/10-5 systems only (that have a median inter-optode distance of 36mm) to avoid too long distances that cannot provide measurements with a proper signal-to-noise ratio [29]. For PROB, iFMRI and fVASC approaches, we only considered channels with inter-optode distance in the range of 20-45mm for Monte Carlo simulations. The number of channels differed across participants as the inter-optode distance could differ with varying head size/shapes across participants (see Table 3 for participant's cap size).

5. Computation of the sensitivity of a channel to a given ROI

Monte Carlo simulations are used to calculate the fluence distribution produced by a source transmitting light into a highly scattering medium [44]. By taking the product of the source and detector fluence distributions (also known as adjoint field), the photon measurement density function can be calculated [83]. This is equivalent to the light sensitivity profiles mentioned earlier. FOLD calculates channel-wise normalized sensitivity profiles from the adjoint field by scaling the adjoint field with the sum of sensitivity of all voxels, so that each voxel represents percentage sensitivity to the whole volume. Then, the sensitivity of a channel to a given ROI is computed as a weighted mean of the voxels within the ROI to the sensitivity of voxels corresponding to the brain (GM and WM):

$$\mathrm{chanSens_{ch}} = 100 \cdot \sum_{\mathrm{k=1}}^{\mathrm{nVoxROI}} \frac{\mathrm{sens_{ch,k}} \cdot \mathrm{w_k}}{\mathrm{brainSens_{ch}} \cdot \mathrm{w'}} \tag{1}$$

where nVoxROI corresponds to the number of voxels comprising the target ROI, $\mathrm{sens_{ch,k}}$ is the normalized sensitivity value for channel *ch* and voxel *k*, $\mathrm{brainSens_{ch}}$ is the normalized sensitivity of channel *ch* of all GM and WM voxels, and *w* corresponds to the value (weight) of the voxel k in the target ROI (adapted from [29]).

The four approaches differed in the nVoxROI and the w parameters. The LIT approach assumed that all voxels belonging to a particular (anatomical) ROI contributed equally to the computation of the sensitivity of a channel to a given ROI and thus all weights were set to one. The PROB approach used probabilistic functional maps that represent the percent overlap of voxels across participants and thus weights ranged between 0 and 100%. As for iFMRI and fVASC, they relied on individual functional activation maps whose weights represent t-statistic values and ranged between 0 and 15.

For the LIT approach, channel sensitivity to a given ROI was computed separately for 10-10 and 10-5 systems as they cannot be computed simultaneously in FOLD. FOLD allows choosing the minimum value of the channel sensitivity to a given ROI to select/discard channels. We set this threshold to 0% in order to select all channels that were somewhat sensitive to the target ROI and combined the list of

output channels for every ROIs that was used for each mental-imagery task. If a channel appeared multiple times for a task, we selected the highest sensitivity value among all instances. As for the remaining three approaches, all channels that were considered for the Monte Carlo simulations together with their associated sensitivity values were selected as input to the next step.

Table S5. Literature review used to select ROIs for the LIT approach.

Author	Title paper	Task			Method
Shergill, Bullmore [84]	A functional study of auditory verbal imagery	IS			fMRI
Baciu, Rubin [85]	fMRI assessment of hemispheric language dominance using a simple inner speech paradigm	IS			fMRI
Fujimaki, Hayakawa [86]	Right-lateralized neural activity during inner speech repeated by cues	IS			fMRI + MEG
Hurlburt, Alderson-Day [87]	Exploring the Ecological Validity of Thinking on Demand: Neural Correlates of Elicited vs. Spontaneously Occurring Inner Speech	IS			fMRI
Girbau [88]	A Neurocognitive Approach to the Study of Private Speech	IS			fMRI, PET, ERP, MEG
Cannestra, Wartenburger [89]	Functional assessment of Broca's area using near infrared spectroscopy in humans	IS			fNIRS
Wan, Hancock [90]	A functional near-infrared spectroscopic investigation of speech production during reading	IS			fNIRS
Zhang, Noah [91]	Signal processing of functional NIRS data acquired during overt speaking	IS			fNIRS
Aziz-Zadeh, Cattaneo [92]	Covert Speech Arrest Induced by rTMS over both Motor and Nonmotor Left Hemisphere Frontal Sites	IS			rTMS
Martin, Brunner [93]	Word pair classification during imagined speech using direct brain recordings	IS			ECOG
Yoo, Fairneny [94]	Brain computer interface using fMRI: spatial navigation by thoughts	IS	MC		fMRI
Sereshkeh, Yousefi [36]	Online classification of imagined speech using functional near-infrared spectroscopy signals	IS			fNIRS + EEG
Herff, Heger [95]	Cross-Subject Classification of Speaking Modes Using fNIRS	IS			fNIRS
Morin and Michaud [96]	Self-awareness and the left inferior frontal gyrus: Inner speech use during self-related processing	IS			fMRI [review]
Verner, Herrmann [97	Cortical oxygen consumption in mental arithmetic as a function of task difficulty: a near-infrarred spectroscopy approach		MC		fNIRS
Pfurtscheller, Bauernfeind [98]	Focal frontal (de)oxygemoglobin responses during simple arithmetic		MC		fNIRS
Kawashima, Taira [99]	A functional MRI study of simple arithmetic - a comparison between children and adults		MC		fMRI
Rickard, Romero [100]	The calculating brain: an fMRI study		MC		fMRI
Power, Kushki [14]	Automatic single-trial discrimination of mental arithmetic, mental singing and the no-control state from prefrontal activity: toward a three-state NIRS-BCI		MC		fNIRS
Shin, Müller [101]	Near-infrared spectroscopy (NIRS)-based eyes-closed brain-computer interface (BCI) using prefrontal cortex activation due to mental arithmetic		MC		fNIRS
Naito, Michioka [13]	A Communication Means for Totally Locked-in ALS Patients Based on Changes in Cerebral Blood Volume Measured with Near-Infrared Light		MC		fNIRS
Weyand and Chau [102]	Correlates of Near-Infrared Spectroscopy Brain–Computer Interface Accuracy in a Multi-Class Personalization Framework	WG	MC		fNIRS
Bauernfeind, Leeb [103]	Development, set-up and first results for a one-channel near-infrared spectroscopy system		MC		fNIRS
Ogata, Mukai [104]	A Study on the Frontal Cortex in Cognitive Tasks using Near-Infrared Spectroscopy	WG	MC		fNIRS
Utsugi, Obata [105]	Development of an Optical Brain-machine Interface	WG	MC		fNIRS
Ang, Juanhong [106]	Extracting and selecting discriminative features from high density NIRS-based BCI for numerical cognition		MC		fNIRS

Schudlo, Power [107]	Dynamic topographical pattern classification of multichannel prefrontal NIRS signals		MC		fNIRS
Schudlo and Chau [108]	Dynamic topographical pattern classification of multichannel prefrontal NIRS signals: II. Online differentiation of mental arithmetic and rest		MC		fNIRS
Arsalidou and Taylor [109]	Is 2+2=4? Meta-analyses of brain areas needed for numbers and calculations		MC		FMRI [meta-analysis]
Hamada, Matsuzawa [48]	Comparison of brain activity between motor imagery and mental rotation of the hand tasks: a functional magnetic resonance imaging study			MR	fMRI
Kawamichi, Kikuchi [52	Distinct neural correlates underlying two- and three-dimensional mental rotations using three-dimensional objects			MR	fMRI
Shimoda, Takeda [56]	Cerebral laterality difference in handedness: A mental rotation study with NIRS			MR	fNIRS
Harris and Miniussi [49]	Parietal lobe contribution to mental rotation demonstrated with rTMS			MR	TMS
Khan and Hong [54	Hybrid EEG-fNIRS-Based Eight-Command Decoding for BCI: Application to Quadrucopter Control	WG	MC	MR	fNIRS and EEG
Tomasino and Gremese [110]	Effects of Stimulus Type and Strategy on Mental Rotation Network: An Activation Likelihood Estimation Meta-Analysis			MR	PET + FMRI [meta-analysis]
Herff, Heger [50]	Classification of mental tasks in the prefrontal cortex using fNIRS	WG	MC	MR	fNIRS
Khalaf, Sejdic [53]	Towards optimal visual presentation design for hybrid EEG—fTCD brain–computer interfaces	WG		MR	EEG + fTCD
Qureshi, Naseer [111]	Enhancing Classification Performance of Functional Near-Infrared Spectroscopy- Brain–Computer Interface Using Adaptive Estimation of General Linear Model Coefficients			MR	fNIRS
Hwang, Lim [51]	Evaluation of various mental task combinations for near-infrared spectroscopy-based brain-computer interfaces		MC	MR	fNIRS
Roberts and Bell [55]	Two- and three-dimensional mental rotation tasks lead to different parietal laterality for men and women			MR	EEG
Tagaris, Richter [57]	Functional magnetic resonance imaging of mental rotation and memory scanning: a multidimensional scaling analysis of brain activation patterns			MR	fMRI
Alivisatos and Petrides [46]	Functional activation of the human brain during mental rotation			MR	PET
Friedrich, Neuper [47]	Whatever Works: A Systematic User-Centered Training Protocol to Optimize Brain-Computer Interfacing Individually	WA	MC	MR	EEG

*** WG: word generation; WA: word association*

Table S6. Selected regions of interest for the LIT-based approach.

Inner Speech (IS)	Mental Calculation (MC)	Mental Rotation (MR)
L | Inferior Frontal Gyrus (p. Opercularis)	*L-R | Middle Frontal Gyrus*	*L-R | Superior Parietal Lobule*
L | Inferior Frontal Gyrus (p. Triangularis)	*R | Angular Gyrus*	*L-R | Inferior Parietal Lobule*
L | Superior Temporal Gyrus	*L | Superior Frontal Gyrus*	*L | Precentral Gyrus*
L | Supramarginal Gyrus		*L-R | Middle Frontal Gyrus*
L | Rolandic Operculum		*L-R | Middle Occipital Gyrus*
L | Precentral Gyrus		

*** L = left hemisphere; R = right hemisphere*

Optimization of the optode layout

As described in the methods section, the optimization problem was set to two constraints. These lines describe the implications of such constraints. The first constraint stated that the inter-optode distance was limited to the 25-40mm range. We used individual inter-optode distance measures derived from the neuronavigation session for this step. This was applied to all four layouts (thus including the layout based on the LIT approach). FOLD toolbox (used for LIT approach) uses near-neighbor channels with a median inter-optode distance of all channels to be 36mm, in MNI space[29]. We used this additional information to ensure that (1) all channels were in the 25-40mm range in the subject-specific space, and that (2) the signal-quality standards for all approaches were as similar as possible.

The second constraint stated that the optode layout for each approach consisted of two channels that shared a common detector (thus including three optodes per approach). Since we do not distinguish between sources and detectors in the Monte Carlo simulations, the sensitivity of the channel will remain the same whether one considers optode X a source and optode Y a detector, or vice-versa. However, due to the second constraint, the algorithm may select a different channel pair that maximizes the total sensitivity to the ROI depending on which optode is considered a source or a detector. To ensure that as many candidate channels as possible were considered during the optimization approach, the optimization problem was solved twice: (1) using the original channel pool that consisted of all optode pairs that were considered for the Monte Carlo simulations (on average, there were 633.25 channels [SD=44.13] across participants); (2) considering their swapped versions (sources were considered detectors and vice-versa).

Mental-imagery task-pair selection process for fNIRS session

We carried out the task-pair selection at the individual subject level. For that, we first calculated the number of overlapping channels across all four layouts for each mental-imagery task, and selected the two tasks with the least number of overlapping channels. We computed the center of gravity (COG) for all four layouts per mental-imagery task in case this approach was not sufficient to select the two

tasks (indicated with *?* in Table S7). The mental tasks with the least number of overlapping channels and highest distance between them were the selected tasks.

Table S7. Summary of the steps involved in selected the task-pair for each participant.

	1st criterion: min # of overlapping channels between tasks			Result →		*2nd criterion: max distance between tasks*			Result →		*Change selected task pair if original combination proofs incompatible*		
	**Overlapping channels**			Selected task pair		**Distance between COGs (mm)**			Selected task pair		Conflict?	Selected task pair	
	IS	MC	MR			**IS**	**MC**	**MR**					
**P01**	2	2	0	?	MR	27,50	45,50	137,35	MC	MR	No	MC	MR
**P02**	0	0	2	IS	MC	136,18	173,11	106,48	IS	MC	No	IS	MC
**P03**	1	2	1	IS	MR	68,60	157,62	193,71	IS	MR	No	IS	MR
**P04**	2	1	0	MC	MR	107,60	187,46	195,29	MC	MR	No	MC	MR
**P05**	1	2	0	IS	MR	132,13	59,89	87,30	IS	MR	**YES**	IS	MC
**P06**	2	2	0	?	MR	71,25	120,98	206,58	MC	MR	No	MC	MR
**P09**	2	2	0	?	MR	143,84	152,83	212,09	MC	MR	No	MC	MR
**P10**	2	2	2	?	?	81,36	192,21	175,89	MC	MR	No	MC	MR
**P11**	3	2	2	MC	MR	75,69	74,12	123,46	MC	MR	No	MC	MR
**P14**	2	2	0	?	MR	65,48	145,50	226,89	MC	MR	No	MC	MR
**P15**	2	2	2	?	?	72,95	155,34	154,82	MC	MR	No	MC	MR
**P16**	1	1	2	IS	MC	107,86	220,43	164,65	IS	MC	**YES**	MC	MR
**P17**	0	2	0	IS	MR	112,77	163,57	237,51	IS	MR	**YES**	MC	MR
**P19**	1	2	2	IS	?	213,88	147,34	150,48	IS	MR	**YES**	MC	MR
**P20**	3	2	0	MC	MR	48,38	138,12	189,18	MC	MR	No	MC	MR
**P21**	2	2	1	?	MR	201,47	139,88	207,34	IS	MR	No	IS	MR

Subject-specific optode layout for the selected mental-imagery task pair

It could be that the source-detector arrangement was not compatible across layouts (within or across mental tasks), since a source in a given channel cannot be a detector in another one (or *vice versa*). To account for such possibility, we first swapped sources for detectors in the problematic spots. This step solved the compatibility problem in all but four participants (P05, P16, P17 and P19). For these participants, using a different mental-imagery task combination solved the issue (see Supplementary Table S5). Since the fNIRS system used in this study uses lighter wires for sources than for detectors, we rearranged sources and detector positions in all participants (when possible) to maximize the number of sources while preserving the channels defined in the optimization step. It is important to note that each participant ended up with a unique optode layout, with a varying number of optodes (see Table S8 and Fig. S6).

Table S8. Subject-specific fNIRS-session summary and optode-layout information.

Participant ID	Cap Size (cm)	Mental tasks		# Runs	# Optodes S | D		# NDC channels	IOD (mm) Mean	| Std. dev.
**P01**	56	MC	MR	6	8	7	13	29,92	2,60
**P02**	56	IS	MC	6	8	9	16	29,00	3,48
**P03***	58	IS	MR	6	8	6	16	34,06	6,84
**P04**	56	MC	MR	6	8	10	15	31,60	4,79
**P05**	60	IS	MC	6	8	4	12	30,67	4,52
**P06**	56	MC	MR	6	8	9	14	31,07	4,20
**P09**	60	MC	MR	6	8	5	9	29,33	4,42
**P10**	56	MC	MR	6	8	7	10	30,40	4,12
**P11**	56	MC	MR	6	8	4	12	30,17	3,19
**P14**	58	MC	MR	5	8	6	11	31,09	3,14
**P15**	58	MC	MR	5	8	5	10	31,70	4,64
**P16**	58	MC	MR	6	8	8	12	31,58	3,42
**P17**	56	MC	MR	6	8	5	11	30,18	4,33
**P19**	56	MC	MR	6	8	7	12	29,83	2,79
**P20**	56	MC	MR	6	8	10	15	31,13	4,50
**P21***	54	IS	MR	6	8	10	14	29,07	3,25

Note: P03 and P21 were excluded from data analysis (see participant exclusion criteria)
Abbreviations: NDC= normal distance channels; IOD= inter-optode distance; MC = mental-calculation; MR= mental-rotation.

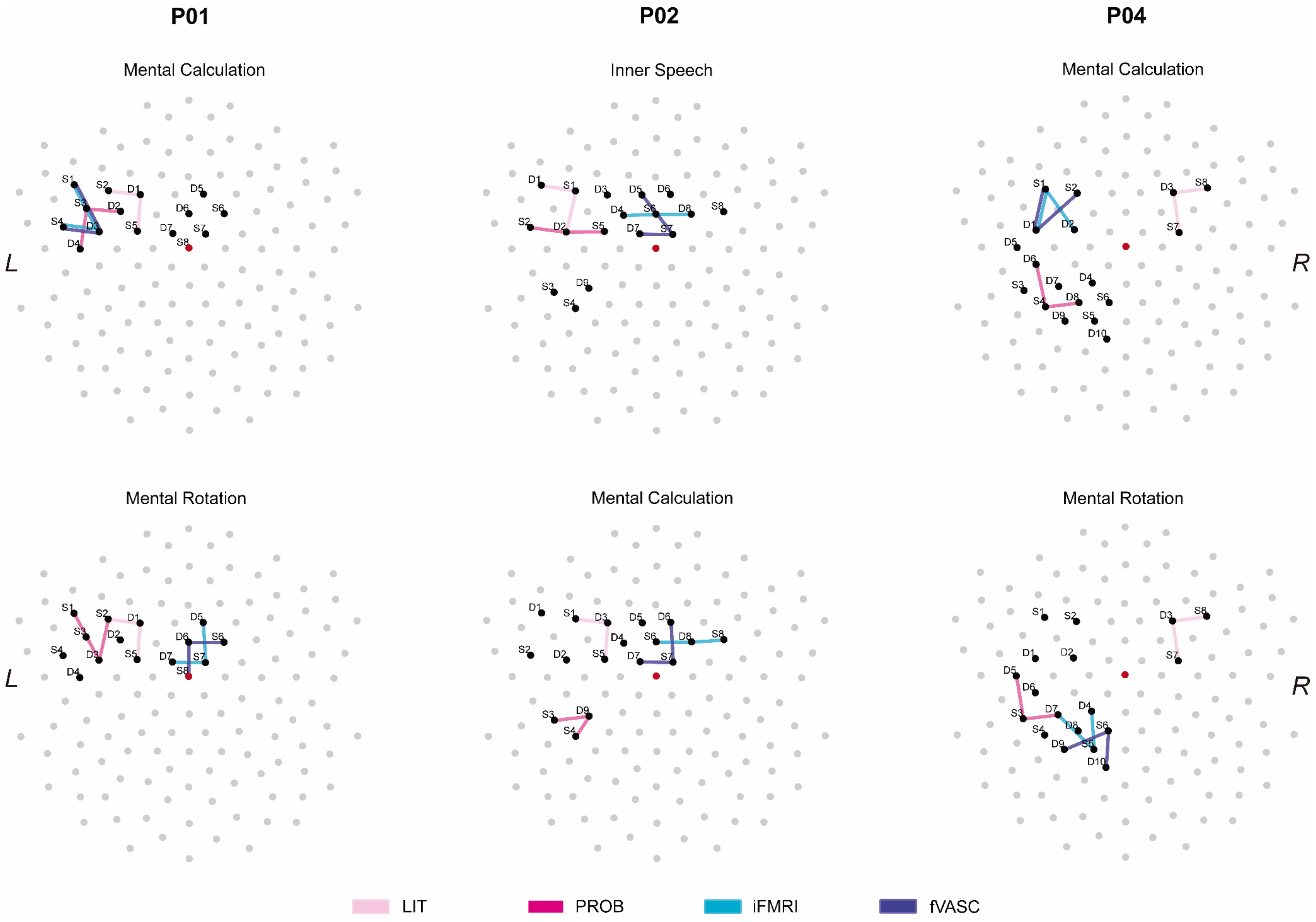
P01
P02
P04
Mental Calculation
Inner Speech
Mental Calculation
Mental Rotation
Mental Calculation
Mental Rotation
L
R
LIT
PROB
iFMRI
fVASC

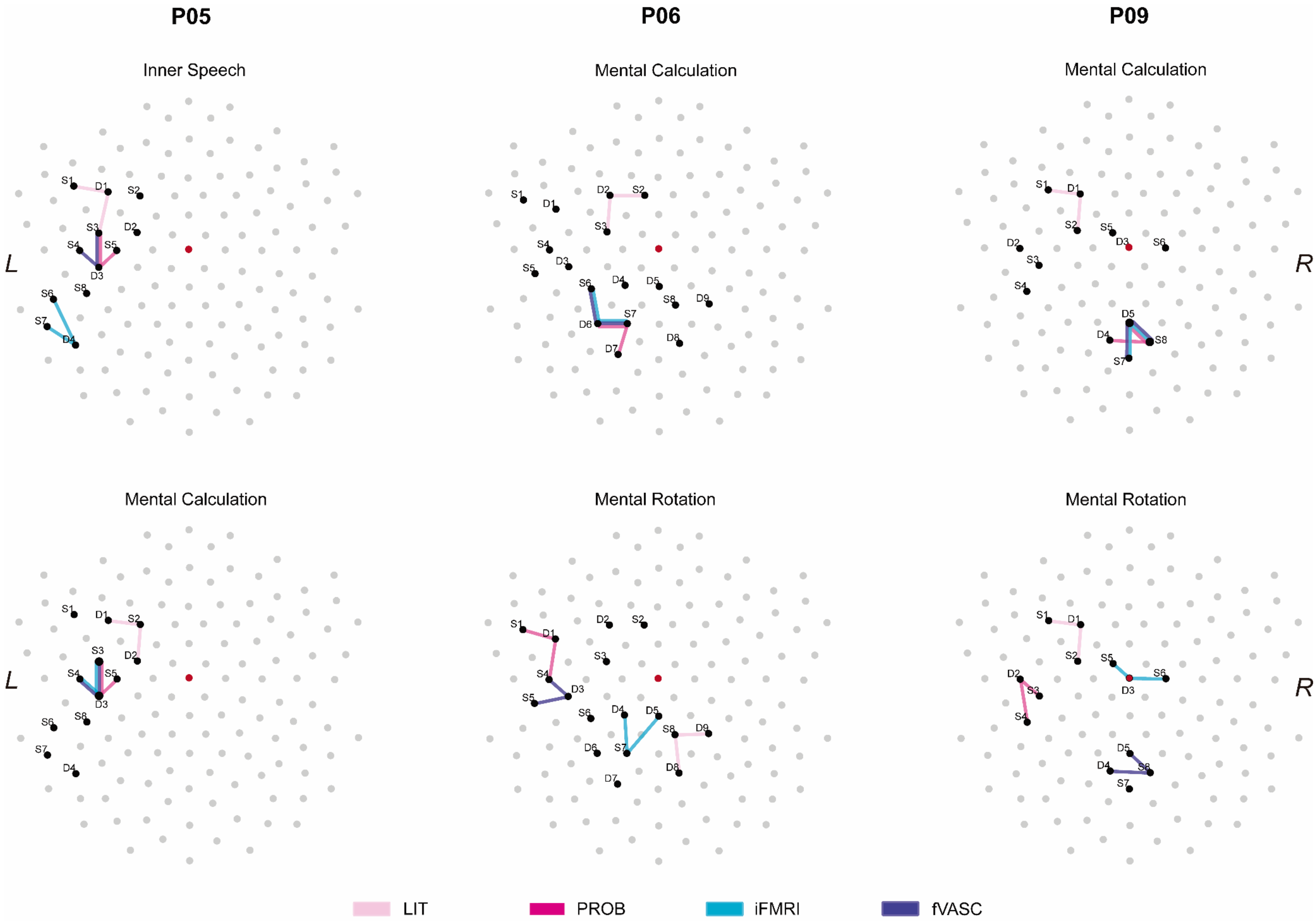

P05
P06
P09
Inner Speech
Mental Calculation
Mental Calculation
Mental Calculation
Mental Rotation
Mental Rotation
L
R
LIT
PROB
iFMRI
fVASC

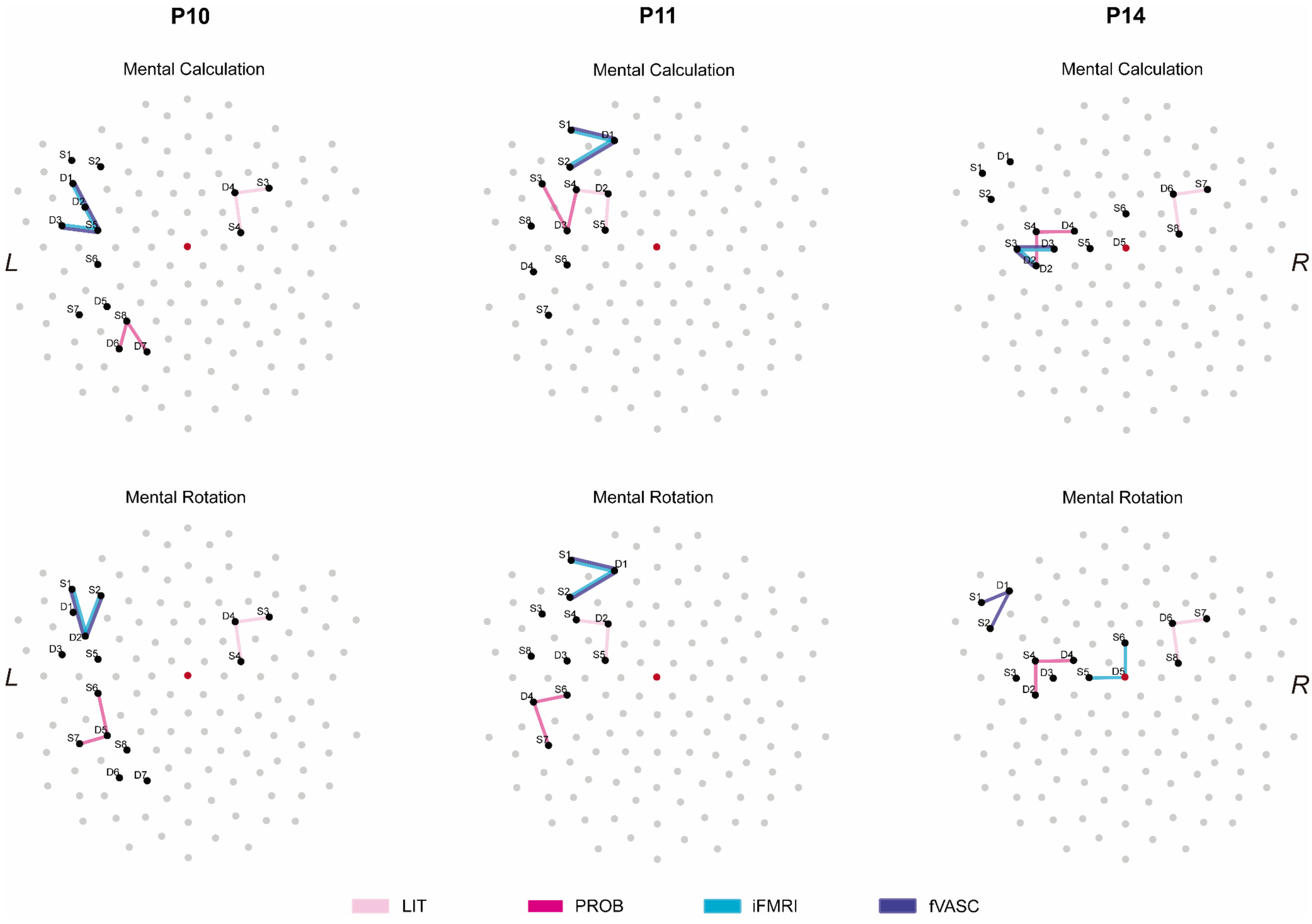

P10
P11
P14
Mental Calculation
Mental Calculation
Mental Calculation
Mental Rotation
Mental Rotation
Mental Rotation
L
R
LIT
PROB
iFMRI
fVASC

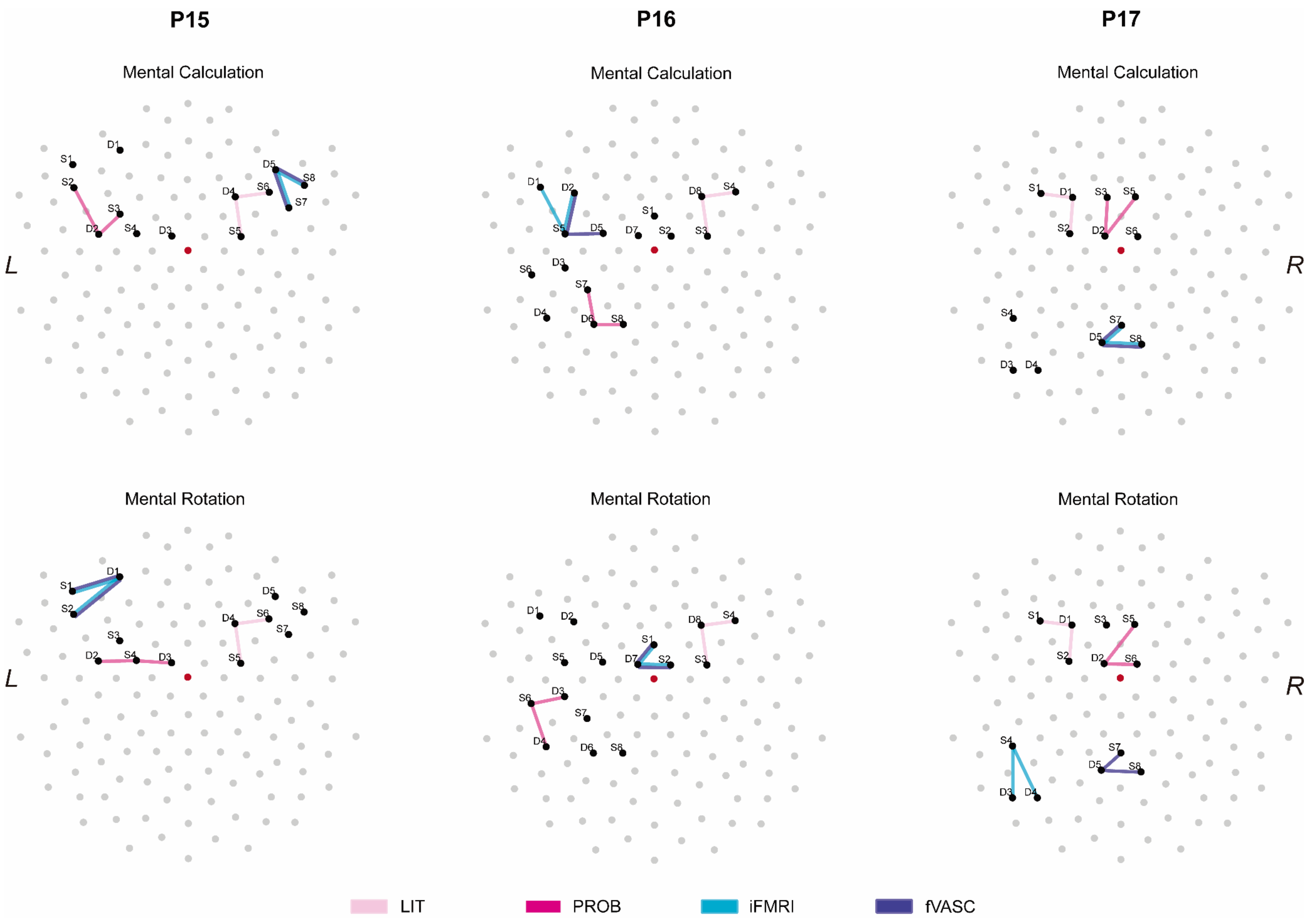

P15
P16
P17
Mental Calculation
Mental Calculation
Mental Calculation
Mental Rotation
Mental Rotation
Mental Rotation
L
R
L
R
LIT
PROB
iFMRI
fVASC

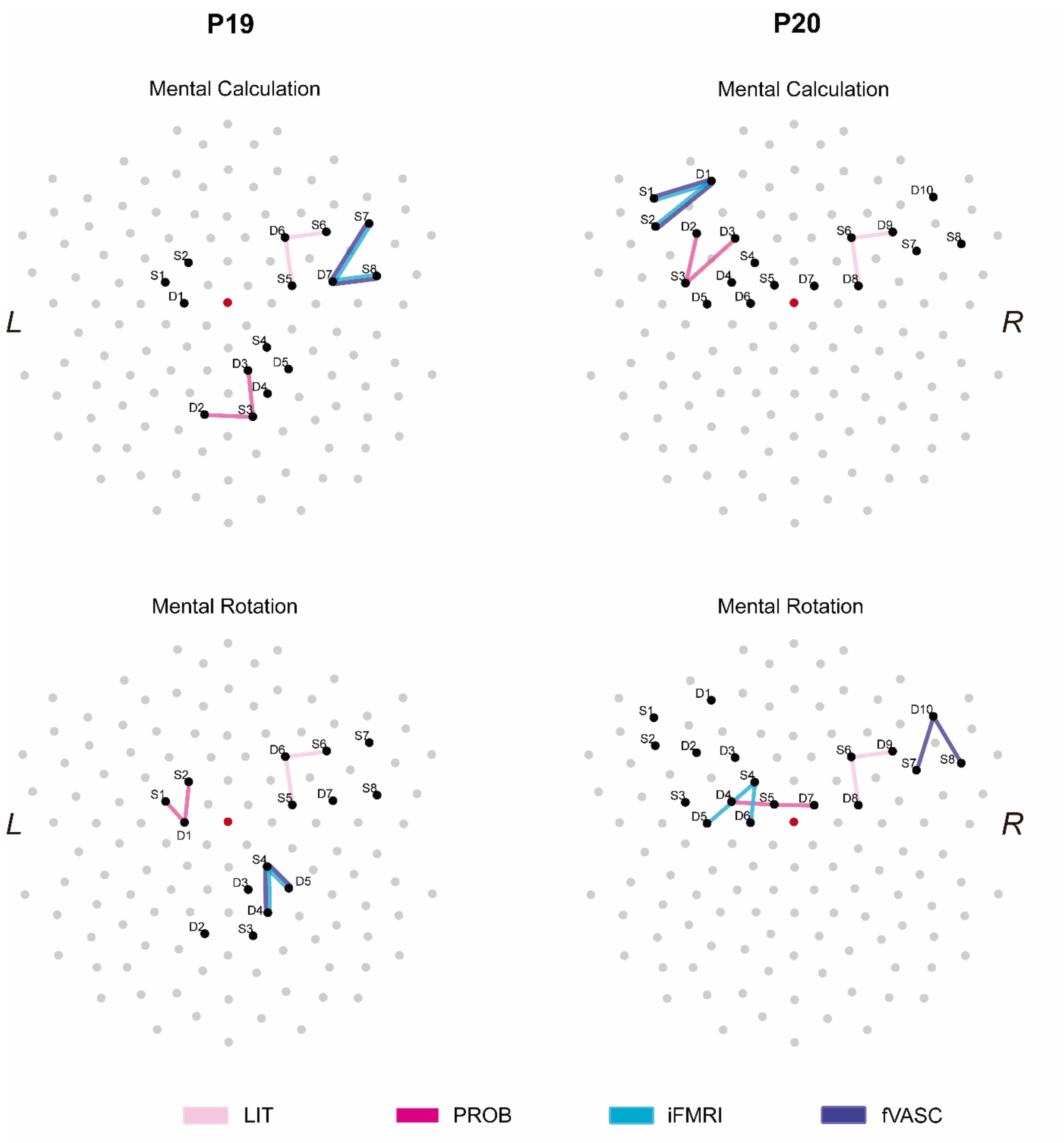

Fig. S6. Subject- and approach-specific optode layouts for each mental-imagery task (top view). Line colors represent the approach used to generate a given layout. ‘S’ represents sources and ‘D’ represents detectors. Cz location is marked in red.

A.3 FNIRS data analysis

Participant exclusion, data quality and presence of motion artifacts assessment

P03 and P21, were excluded from subsequent analysis for different reasons. The optode layout for P03 was created based on a different inter-optode distance range criterion than the rest of the participants (25-45mm vs. 25-40mm). This is because P03 was the first participant who participated in the fNIRS session and the original inter-optode distance range was expected to provide reasonable signal quality. However, this range proved to be suboptimal as four NDC and three SDC did not survive the coefficient of variation threshold (CV < 7.5%), a metric used to estimate the signal-to-noise ratio for each channel [112]. Given the restricted number of channels comprising each layout, we created the layouts for the rest of the participants using a more conservative inter-optode distance range criterion (25-40mm range, see Sec. A.4, *Optimization of the optode layout*) to ensure that all (or as many as possible) channels survive the CV threshold. Thus, P03 was excluded for comparability reasons. As for P21, the data was corrupted and could not be retrieved.

We computed the coefficient of variation (CV) to quantify the signal quality in each channel. Channels with CV >=7.5% were discarded from subsequent analyses. Figure S7 shows the percent of channels that fulfilled the CV criterion for each participant.

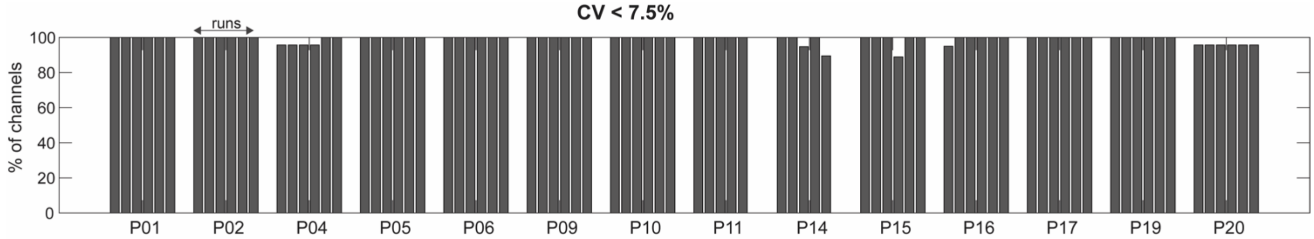

Fig. S7. Signal quality. This figure shows the percent of channels (y-axis) across runs (represented as bars) and participants (x-axis) that survived the CV threshold of 7.5%. Almost all channels met the CV criterion across participants.

We used *hmrMotionArtifactByChannel* for motion correction, with the following parameters: AMPThresh=0.15, tMotion=0.5 and tMask=2. The SDThresh parameter ranged between 8 and 10 across participants. Motion artifact identification was visually assessed by experimenter AB and was manually corrected in case it was necessary. Motion artifacts were divided into spikes and baseline shifts. Baseline shifts were corrected using *hmrSplineInterp* algorithm in Homer2 (p=0.99), while *hmrMotionCorrectWavelet* algorithm in Homer2 (iqr=0.5) was used to correct for the spike artifacts only in the channels where motion artifacts had been detected. The top panel of figure S8 summarizes the detected motion events per channel and run for each participant. The bottom panel provides cumulative motion events across channels and runs for participant P14.

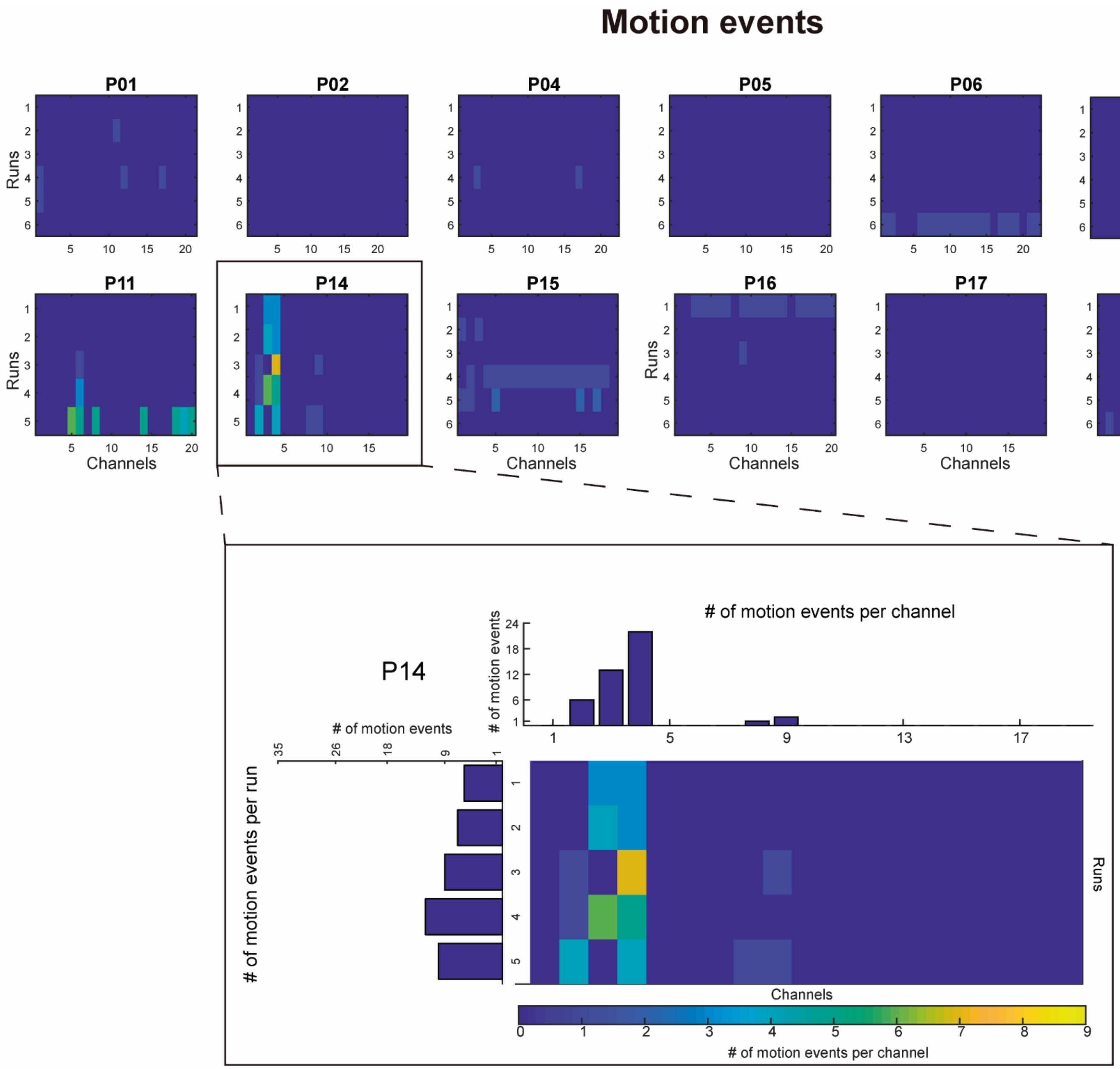

Fig. S8. Motion artifacts. **Top.** Presence of motion artifacts across channels (x-axis in subplot), runs (y-axis in subplot) across participants. The color in each cell indicates the frequency of detected motion artifacts within a run for a given channel. Note that the number of channels and runs were different across participants. **Bottom.** Closer look into the motion artifacts in participant P14. The top histogram shows the cumulative number of motion events across runs for a given channel, while the histogram on the left shows the cumulative motion effects over channels for a given run.

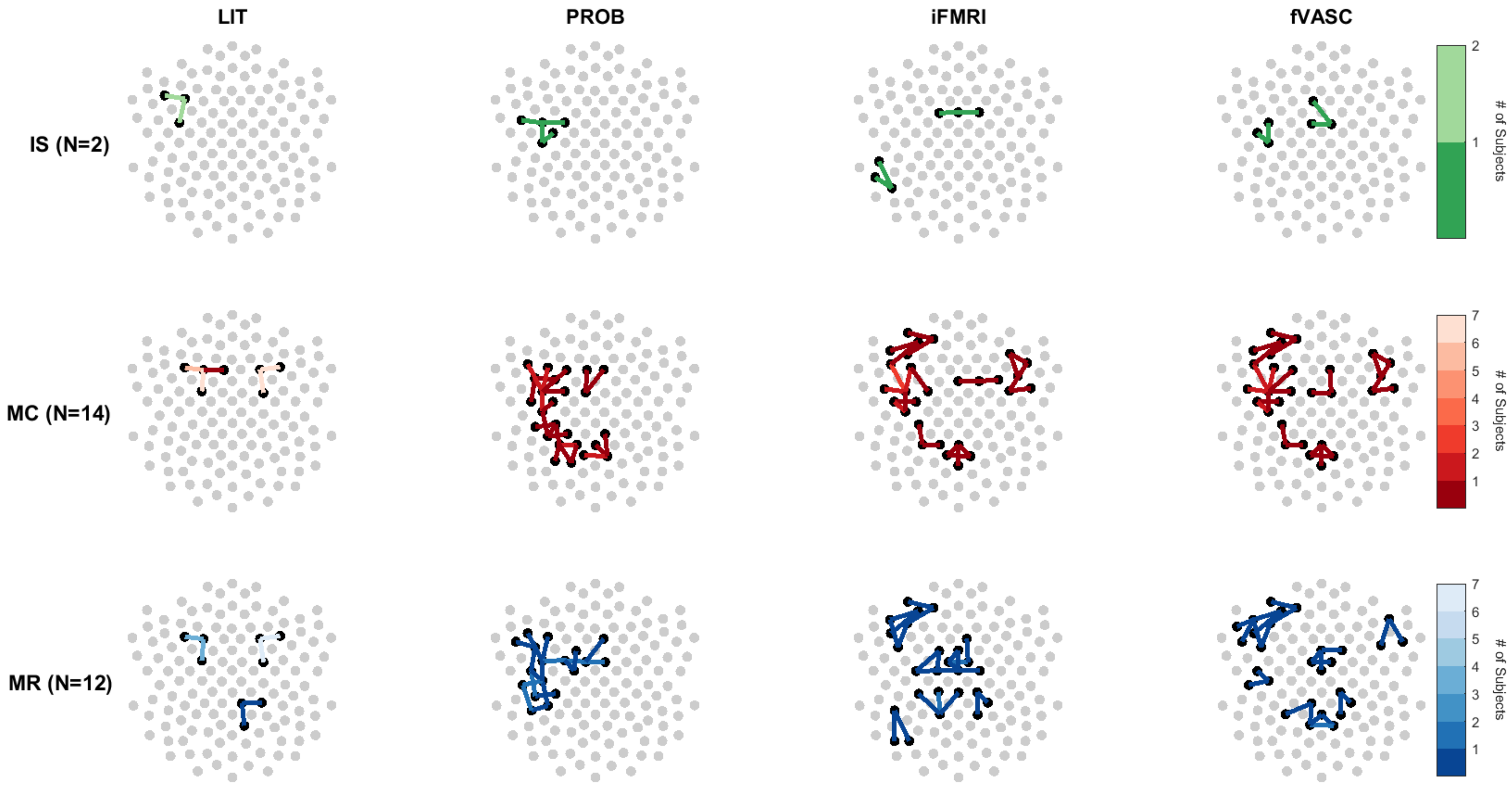

Fig. S9. Top view of the channel frequency maps for each mental-imagery task (rows) and approach (columns). Black dots indicate the locations where optodes were placed, while grey dots represent all 130 locations optodes could be located. Channels are indicated with lines and their colors indicate the number of participants who used a given channel. The selected channels vary considerably across subjects for PROB, iFMRI and fVASC approaches. iFMRI and fVASC approaches show highest similarity and spatial extent.

fNIRS data projection onto cortical surface using inverse distance weighting and comparison with fMRI data

Each fNIRS channel position was defined as the point in the scalp half way between the corresponding source and detector position. The cortical projection of each channel was determined by taking the point in the brain reconstruction closest to the channel position in the scalp. A sphere of radius *r* was centered in the projected cortical point and the voxels inside the sphere that were labeled as GM were assigned a weight depending on how far from the center they were located. The weight (w) was calculated as $1/d^2$, where *d* is the Euclidian distance between the projected point (center of the sphere) and a given voxel inside the sphere. At each cortical vertex *k* inside the sphere, the interpolated fNIRS data was computed as:

$$s(k) = \frac{\sum_{i=1}^{n} w_i * f_i}{\sum_{i=1}^{n} w_i} \quad (5)$$

where *n* is the number of cortical projection points and *f* is the amplitude of the fNIRS channel value. Here we used two cortical projection points as two channels comprised a given layout. The channel-specific amplitude was calculated as the average value of the normalized fNIRS signal (computed as the channel time course divided by its peak value) in the range of 3s after task onset to 5s after task offset. In total, four spheres with varying radii (r = {10, 15, 20, 25} mm) were used.

We used channel-specific projection weights and projection spheres to compute spatially weighted fMRI block averages to assess the temporal correlation between fNIRS and fMRI signals. First, voxels inside the sphere of radius *r* that were labeled as GM were selected and mental imagery-specific events were extracted from each voxel's time courses. Task-specific ROI averages were computed by weighting the contribution of each voxel according to the projection weights. The standard error of the weighted average was estimated using bootstrapping (with 100 resamples and sample size equal to 60% of the initial number of voxels). These steps were repeated for every channel across all layouts in each participant. Finally, the temporal correlations of fNIRS and fMRI block averages were computed using Pearson's correlation. Next to channel-specific projection weights, layout-specific projection weights were also calculated. Their computation differed in that for the latter we used the center of gravity of each layout on the scalp to determine the cortical projection point. Layout projection weights were used to extract the peak and spatially weighted average *t*-estimates of individual fMRI activation of the voxels labeled as GM to assess how well the fNIRS ROIs targeted individual activation maps.

B. Results

B.1 Frequency maps

We computed frequency maps for each mental-imagery task and approach to assess the spatial agreement of the selected channels across participants. The frequency maps shown in Figure S9 indicate that the selected channels varied considerably across subjects for PROB, iFMRI and fVASC approaches. In addition, iFMRI and fVASC approaches (the two most individualized ones) show the highest and most similar spatial extension for MC and MR. It is important to note that the (low)

variability observed in the LIT approach is due to the use of the minimal individual anatomical information during the channel selection step (see Sec. A.4, *Optimization of the optode layout*).

B.2 Effect sizes

Cohen's d was calculated as in [42] using the following formula:(mean(dur)-mean(pre))/std(pre), where *dur* and *pre* represent a section of the task and rest periods, respectively (we used the same temporal window as for the CNR definition, see Sec. 2.2.5). This metric was computed after AR correction.

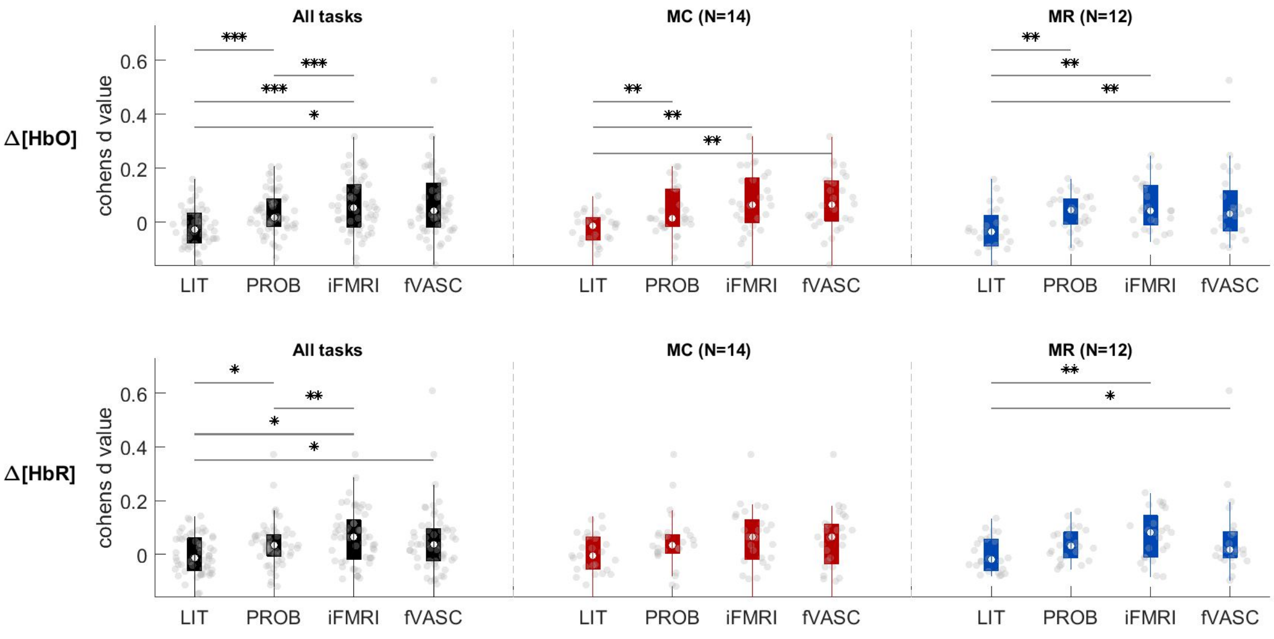

Figure S10. Effect sizes-based group comparison across layouts. Results were evaluated separately for Δ[HbO] (top, in each subplot) and Δ[HbR] (bottom, in each subplot), when all three mental-imagery tasks were considered together as well as separately for MC and MR tasks (left, middle and right column, respectively). LIT performed significantly worse than the PROB, iFMRI and fVASC approaches for both chromophores when all tasks were considered together. A similar pattern was observed for MC and MR tasks for Δ[HbO]. Gray dots represent single-subject CNR/ROI t-statistic values for a given mental-imagery task. Whiskers represent the 1.5 times the inter-quartile range. Significant parwise differences (calculated using Wilcoxon signed-rank test, one-sided and corrected for multiple comparisons) are indicated with asterisks: *** = q[FDR] < 0.001; ** q[FDR] < 0.01;* q[FDR] < 0.05. Abbreviations: MC = mental-calculation; MR= mental-rotation.

B.3 Examples of typical and weak/inverted hemodynamic responses

Figure S11A shows examples of four participants with typical hemodynamic responses (a positive deflection in HbO and a negative deflection in HbR) for a given approach, together with the projected activation on individual cortex reconstructions. Figure S11B shows examples of four participants with weak/inverted hemodynamic responses.

(a) Examples of typical hemodynamic response

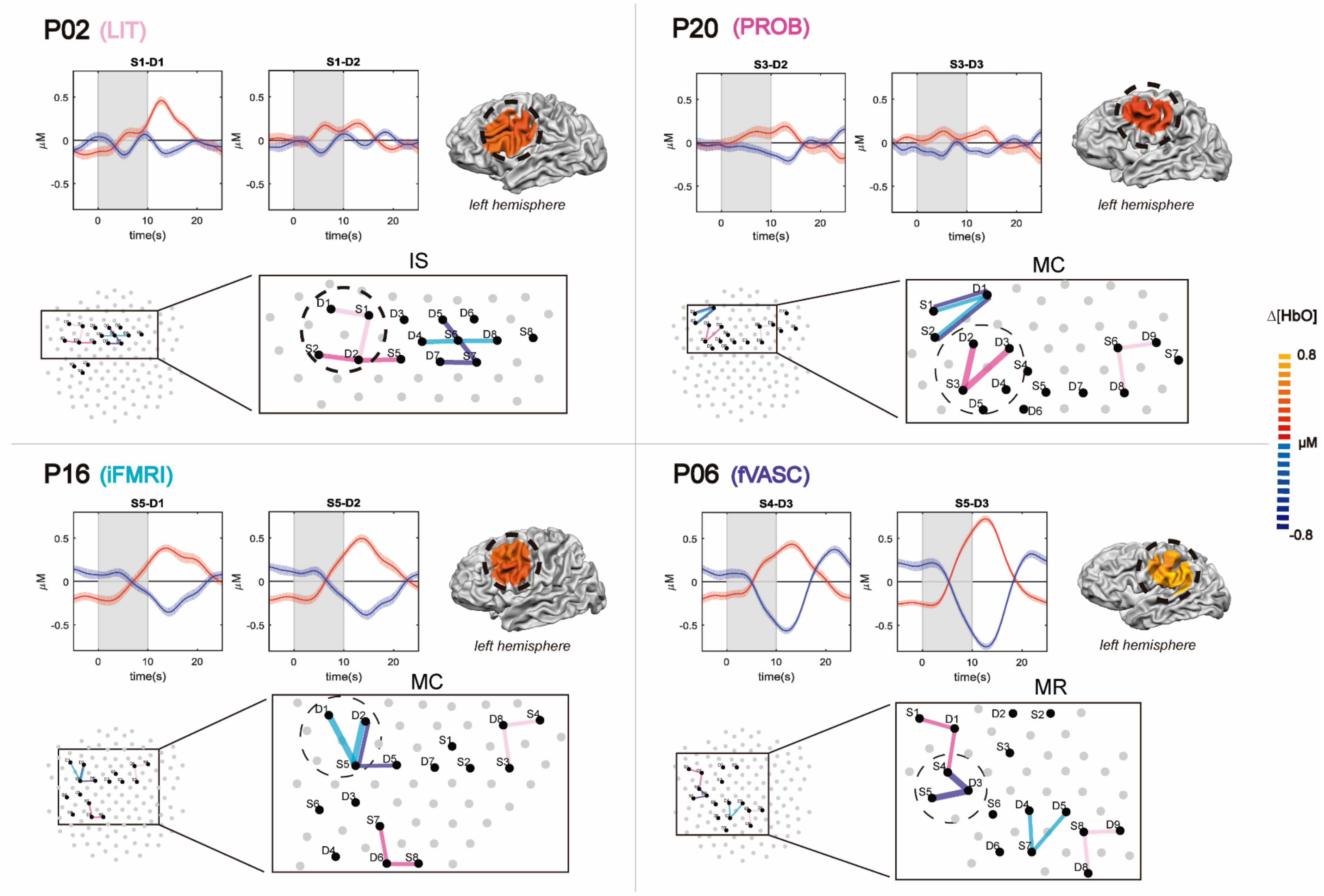

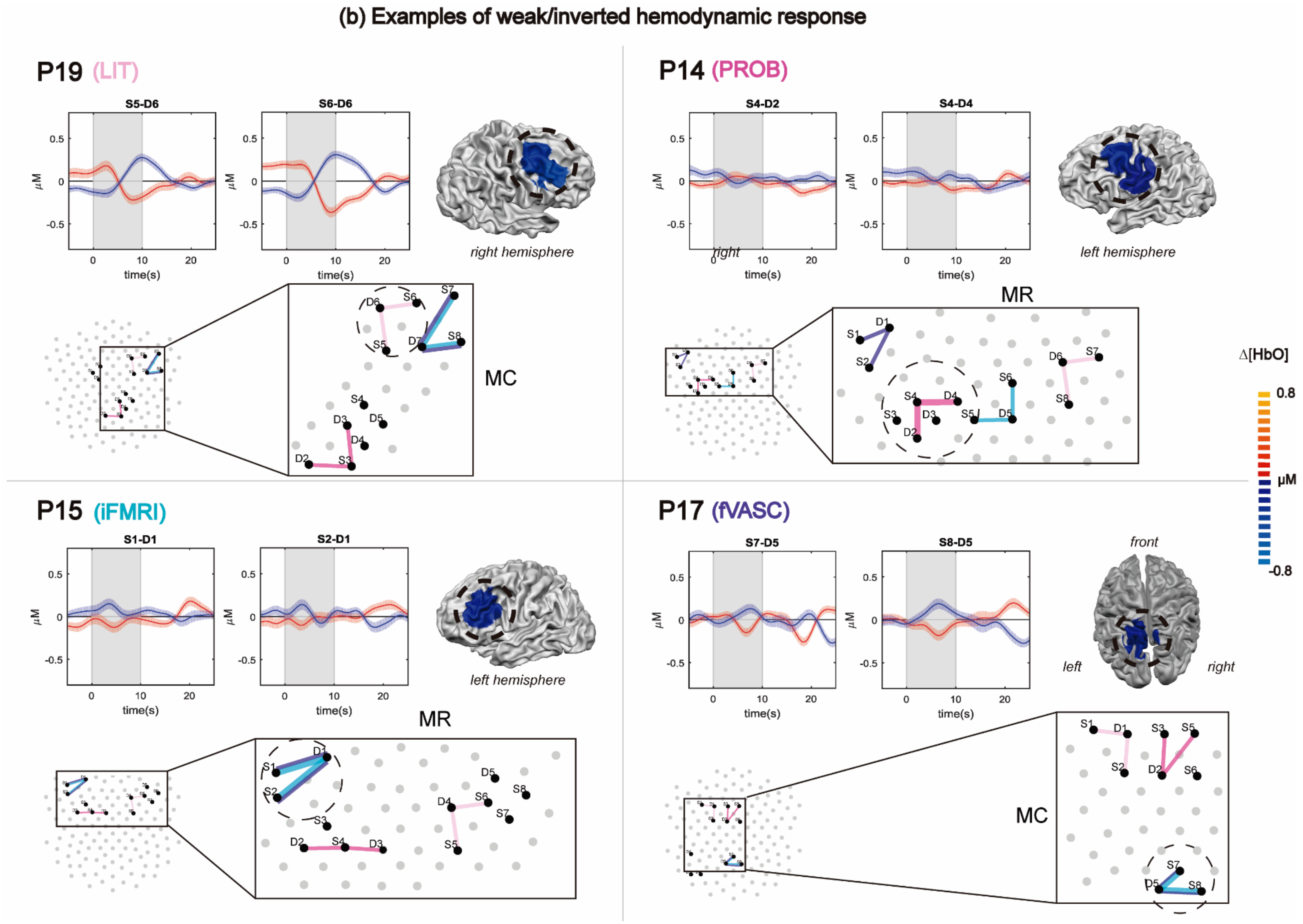

Fig S11. Examples of typical (a) and weak/inverted (b) hemodynamic responses, for every approach-specific layout and mental-imagery tasks. Every plot depicts a different participant. On the top part, fNIRS block averages of the two channels comprising a given layout are shown, with Δ[HbO]signal in red and Δ[HbR]signal in blue. The grey area indicates the onset and duration of the mental-imagery task (MC = Mental Calculation; MR= Mental Rotation). The bottom left plot shows the approach-specific optode layout for the same mental-imagery task as the block averages. The top right plot illustrates the projection of the two fNIRS channels in the individual anatomical data (3D surface reconstructions) for Δ[HbO].

C. Discussion

C.1 Understanding why LIT layout performs worse than the other three layouts

LIT ROIs were transformed to individual subject space and dice coefficients between individual activation maps and aligned LIT ROIs were computed. For comparability reasons, dice coefficients between individual activation maps and probabilistic maps were computed. Figure S12 shows that the average dice coefficients (across participants and tasks) are significantly lower for LIT ROIs than for probabilistic maps ($p<0.001$). Next, we extracted the peak t-values for each modality. For LIT, we extracted the peak t-value of the individual activation maps that was part of the LIT ROI. Peak t-values across participants and tasks are significantly lower for LIT ROI ($p<0.001$).

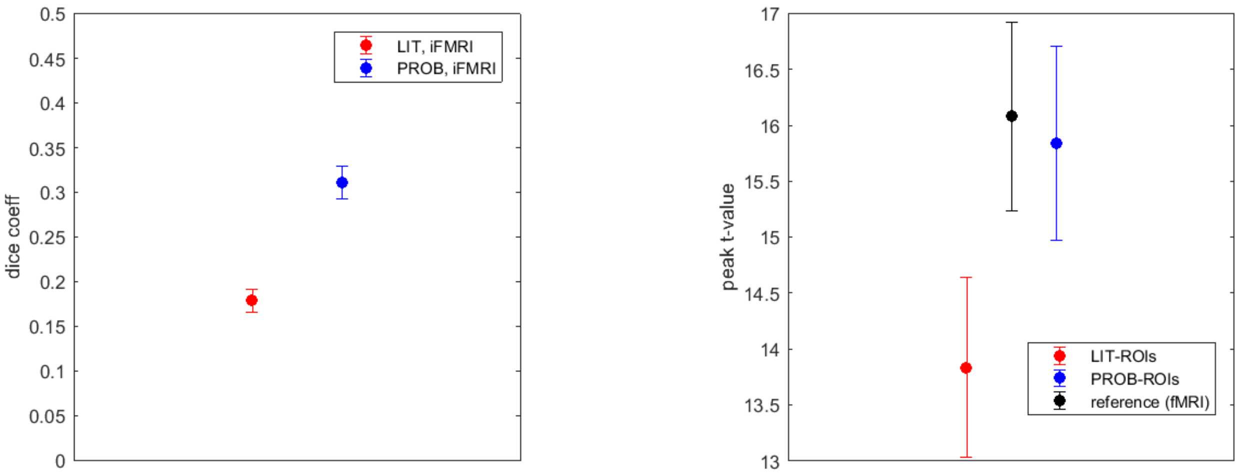

Figure S12. Comparison of LIT, PROB and iFMRI activation maps when LIT ROIs are transformed to individual subject space. Left. Dice coefficient between LIT and iFMRI (red) is significantly lower than compared to the dice coefficient between PROB and iFMRI maps (blue). **Right**. The average peak values for LIT-ROIs are significantly lower than for PROB or iFMRI-based ROIs. Error bars represent the standard error of the mean.

C.2 Correspondence between fMRI and fNIRS block averages

Figure S13 shows the correspondence between fMRI and fNIRS block averages calculated from channels placed according to the four approaches, for participants P01 and P16 during MR and MC tasks, respectively. We used channel-specific projection weights and projection spheres to compute spatially weighted fMRI block averages of the regions where the fNIRS signal most likely originated (see Sec. A.3 for details). The figure shows that channels placed based on approaches that use more individualized information show a better agreement with fMRI block averages. In addition, channels in close proximity can capture considerably different responses (see P01).

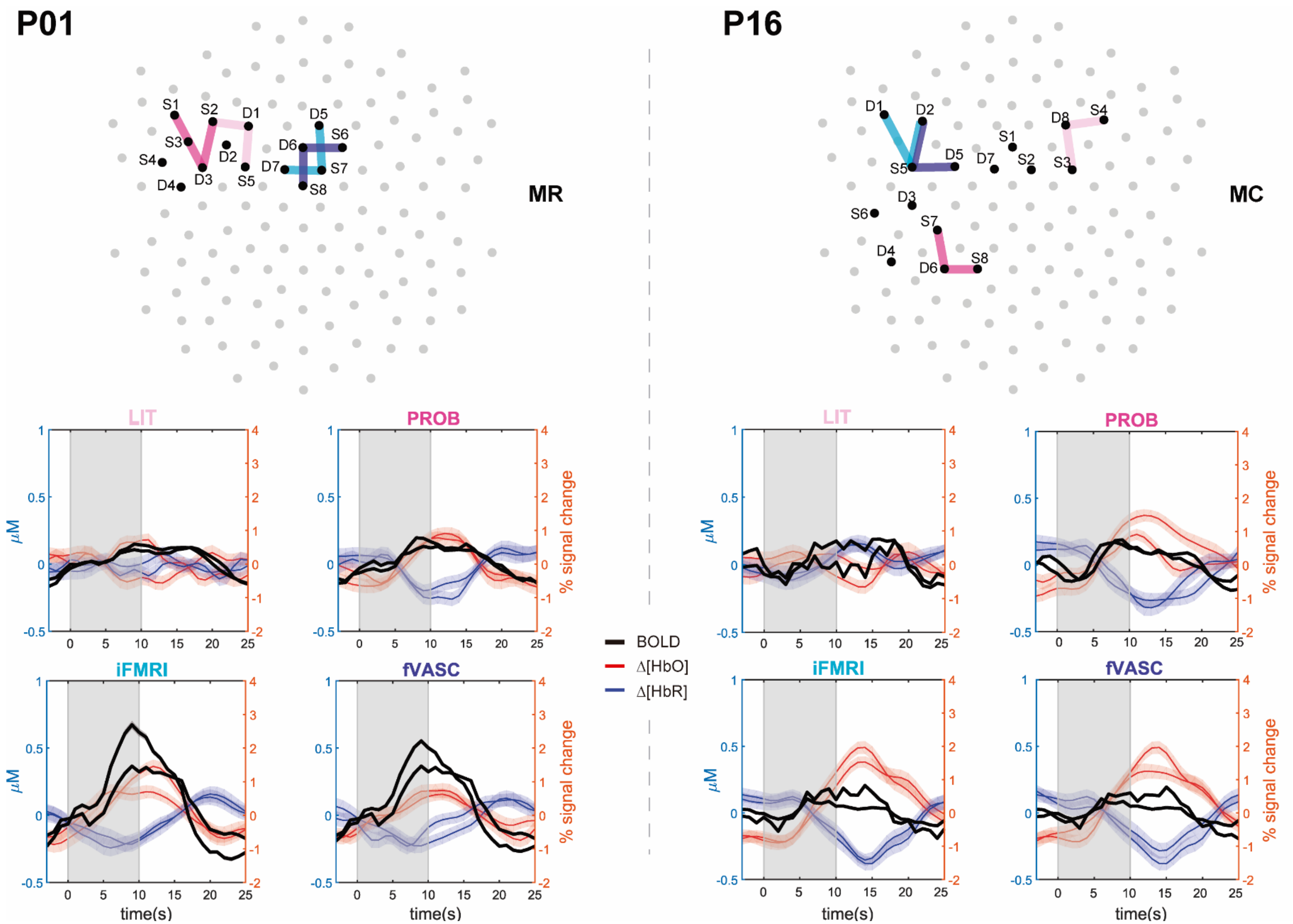

Fig. S13. Correspondence between fMRI and fNIRS block averages across different approaches in participants P01 (left) and P16 (right) for one of the mental-imagery tasks they performed (mental rotation [MR] and mental calculation [MC], respectively). The y-axis on the left represents concentration changes in μM for Δ[HbO] (red) and Δ[HbR] (blue) data, while the y-axis on the right represents the percent signal change for the fMRI time course (BOLD response, black line). The 0 value in the x-axis represents the task onset time (in s) and the gray area depicts the task duration. fNIRS time courses were normalized by the peak value before computing the block averages. The fNIRS block averages derived from the more individualized approaches show better agreement with fMRI data.
